# The function of GPCRs in different bone cells

**DOI:** 10.7150/ijbs.113585

**Published:** 2025-07-24

**Authors:** Yan Zhang, Nai-Ning Wang, Zi-Han Qiu, Jia-Hao Wang, Wen-Na An, Li-Dan Shi, Fei Chen, Da-Jin Zhang, Si-Yue Wang, Tie-Lin Yang, Shou-Ye Hu, Yan Guo

**Affiliations:** 1 Key Laboratory of Biomedical Information Engineering of Ministry of Education, Key Laboratory of Biology Multiomics and Diseases in Shaanxi Province Higher Education Institutions, and Biomedical Informatics & Genomics Center, School of Life Science and Technology, Xi'an Jiaotong University, Xi'an, Shaanxi, 710049, China.; 2 Department of Joint Surgery, Honghui Hospital, Xi'an Jiaotong University, Xi'an, Shaanxi, 710054, China.; 3 Department of Orthopedics, The First Affiliated Hospital of Xi'an Jiaotong University, Xi'an, Shaanxi, 710061, China.

**Keywords:** GPCR, MSC, osteoblast, osteoclast, chondrocyte

## Abstract

G protein-coupled receptors (GPCRs) are recognized as critical therapeutic targets in bone disorders, owing to their multifaceted regulatory roles across diverse bone cell lineages. This review systematically catalogs GPCR expression and functional heterogeneity in key bone cells: 12 GPCRs in mesenchymal stem cells (MSCs) orchestrate lineage specification; 21 GPCRs in osteoblasts/osteocytes mediate matrix mineralization and mechanotransduction; 23 GPCRs in macrophages/osteoclasts regulate inflammatory bone resorption; 31 GPCRs in chondrocytes govern endochondral ossification and osteoarthritis pathogenesis; and 8 GPCRs in other cell types modulate bone-related physiological processes. By integrating canonical signaling axes—cAMP/PKA-dependent transcriptional networks, PLC-β/IP3-driven calcium signaling, and NF-κB-modulated immuno-skeletal interactions—we elucidate how GPCRs dynamically coordinate cellular plasticity to maintain skeletal homeostasis. This work establishes a multidimensional research framework integrating historical context, molecular mechanisms, and cutting-edge methodologies to advance GPCR-targeted therapies for bone-related diseases. Moreover, this review provides insights for clinical translation, including biased agonism and allosteric modulation precision strategies to restore skeletal equilibrium in osteoporosis, arthritis, and regenerative medicine.

## Introduction

Bone modeling is a dynamic and continuous process fundamental for maintaining skeletal integrity and facilitating repair throughout an individual's lifetime. This intricate process hinges on a delicate interplay among multiple cells, governed by a sophisticated network of molecular interactions. Among the pivotal regulators of this process are G protein-coupled receptors (GPCRs), a family of transmembrane proteins that act as critical sensors of extracellular cues and translators of intracellular responses 1-3. GPCRs play indispensable roles in transducing diverse extracellular signals into intracellular responses across various physiological systems.

Ubiquitously expressed across tissues and cells, GPCRs have long been key targets for drug development, accounting for approximately 33% of currently marketed drugs 4, 5. Within skeletal biology, GPCRs directly modulate the functions of mesenchymal stem cells (MSCs), osteoblasts, osteocytes, osteoclasts, chondrocytes and other bone cells, thereby influencing bone metabolism and homeostasis. They have emerged as a significant target family for the treatment of bone-related diseases. Notable examples include the Parathyroid hormone (PTH) receptor, whose agonist abaloparatide promotes osteoblast differentiation while suppressing osteoclast activity, and teriparatide (a recombinant human PTH 1-34 fragment), which inhibits bone resorption, stimulates bone formation, and is approved for treating osteoporosis in postmenopausal women, old men, and glucocorticoid-induced cases 6, 7. Additionally, the Calcium-sensing receptor (CaSR) and its agonists (e.g., calcimimetics) enhance extracellular calcium sensitivity, mitigating hypercalcemia-related complications like osteoporosis and extending therapeutic potential to fracture healing and bone tumors 6. These applications demonstrate a pivotal role of GPCRs in skeletal biology and the regulatory mechanisms of bone modeling, and underscore GPCRs' translational relevance in addressing skeletal disorders.

Skeletal homeostasis emerges from the coordinated activities of diverse bone cell populations, with GPCRs orchestrating specialized functions through context-dependent signaling 1, 2, 7. GPCR activity is influenced by age, genetic, and environmental factors, with functional changes directly contributing to bone metabolic imbalances during development, aging, and disease progression. In MSCs, GPCRs couple to G protein subtypes (e.g., Gs, Gq, Gi) to direct lineage commitment toward osteoblasts or chondrocytes. Osteoblasts utilize GPCRs to regulate matrix synthesis and mineralization while secreting paracrine signals that dampen osteoclastogenesis. Conversely, osteoclast-surface GPCRs integrate hormonal and local cues to modulate resorptive activity, whereas osteocytes, as mechanosensors, employ GPCRs to translate mechanical stimuli into adaptive remodeling signals. Macrophages and chondrocytes also leverage GPCRs to mediate inflammatory responses and joint cartilage metabolism, respectively. Dysregulation of these pathways—such as aberrant GPCR signaling—contributes to pathologies like osteoporosis, osteoarthritis, and rheumatoid arthritis. Emerging study highlights GPCRs as both biomarkers and therapeutic targets, with their functional plasticity offering opportunities for cells interventions 3, 5, 8. Understanding the molecular functions and mechanisms underlying GPCR-mediated regulation in different bone cells has profound implications for the development of novel drug targets and therapeutic strategies for bone-related diseases.

Here, we comprehensively summarize the crucial roles and mechanisms of GPCRs in different bone cells, based on emerging evidence from numerous studies. We also establish a multidimensional framework that integrates historical context, molecular mechanisms, and cutting-edge methodologies to advance GPCR research in bone biology. In conclusion, our review consolidates the current understanding of GPCRs in various bone cells and paves the way for the development of novel drug targets and therapeutic strategies for bone-related diseases. As the field of GPCR research continues to evolve, we anticipate that future studies will further elucidate the functional nuances of these receptors in bone metabolism and uncover new therapeutic opportunities for addressing bone disorders.

## Structure and signaling cascade of GPCR

**Figure 1:**
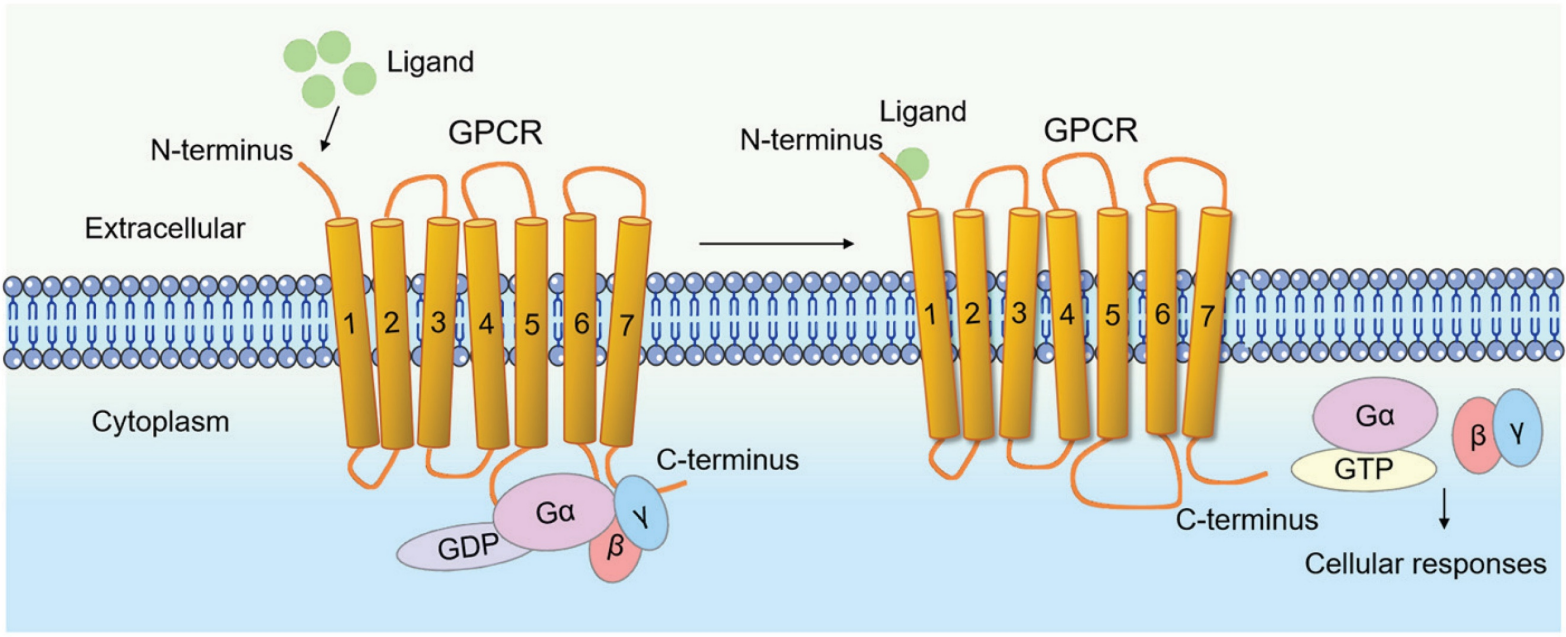
** Schematic representation of the G protein-coupled receptor (GPCR) model.** The hallmark of GPCR receptors is their characteristic seven-transmembrane α-helical structure. The binding site for the G protein (guanylate-binding protein) is located at the C-terminus of the polypeptide chain and on the third intracellular loop between the fifth and sixth transmembrane helices, as counted from the N-terminus. Their activation by external signals triggers a series of biochemical reactions through interactions with different G proteins or arrestins, thereby regulating diverse physiological processes.

GPCRs constitute a large and diverse superfamily of transmembrane proteins, widely distributed and serving as crucial membrane protein receptors 4, 5, 9. The hallmark of GPCRs is their characteristic seven-transmembrane α-helical structure. The binding site for the G protein (guanylate-binding protein) is located at the C-terminus of the polypeptide chain and on the third intracellular loop between the fifth and sixth transmembrane helices, as counted from the N-terminus (Fig. 1).

**Figure 2:**
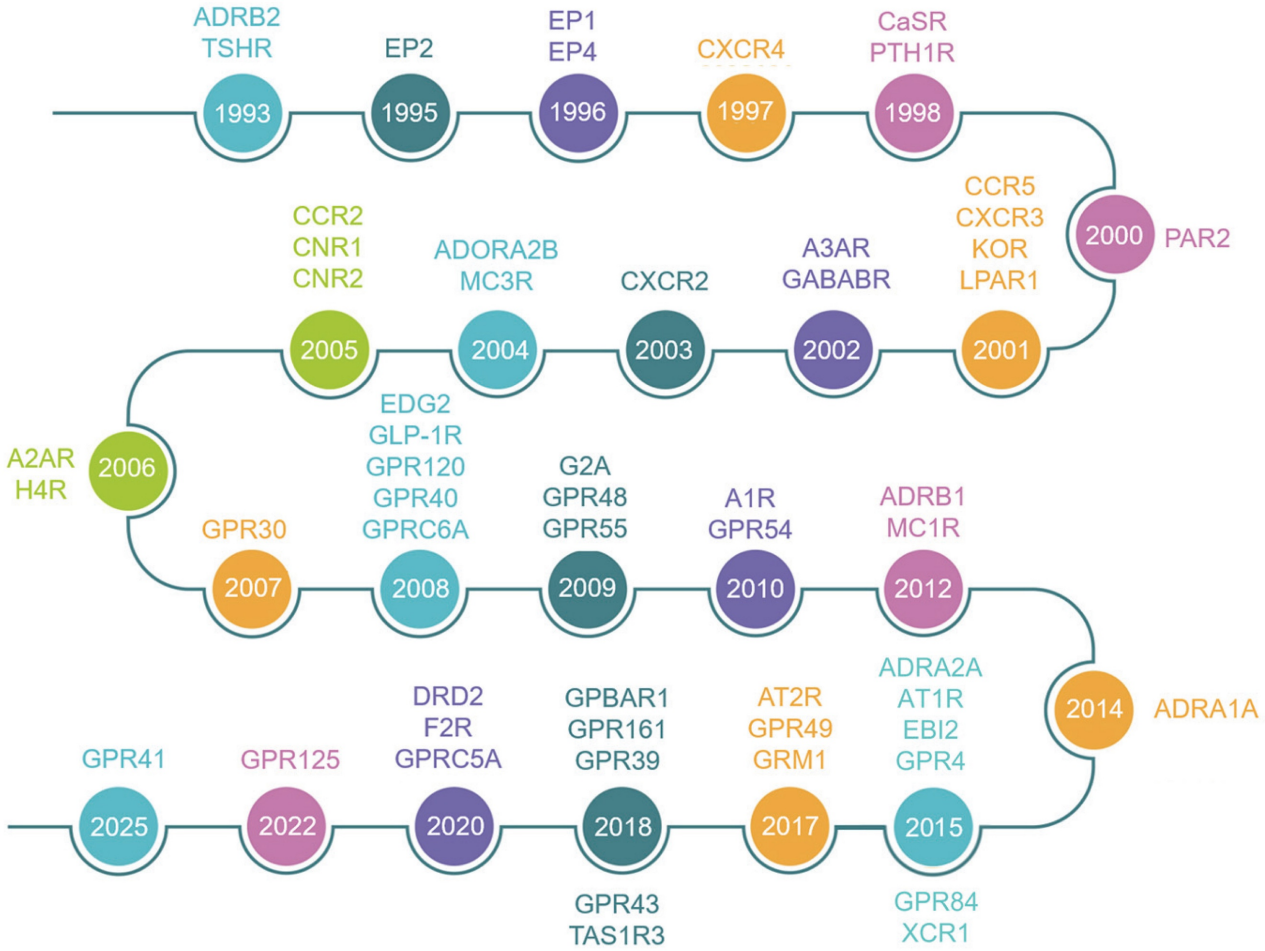
Historical timeline of the major GPCRs discovered in bone biology.

**Figure 3:**
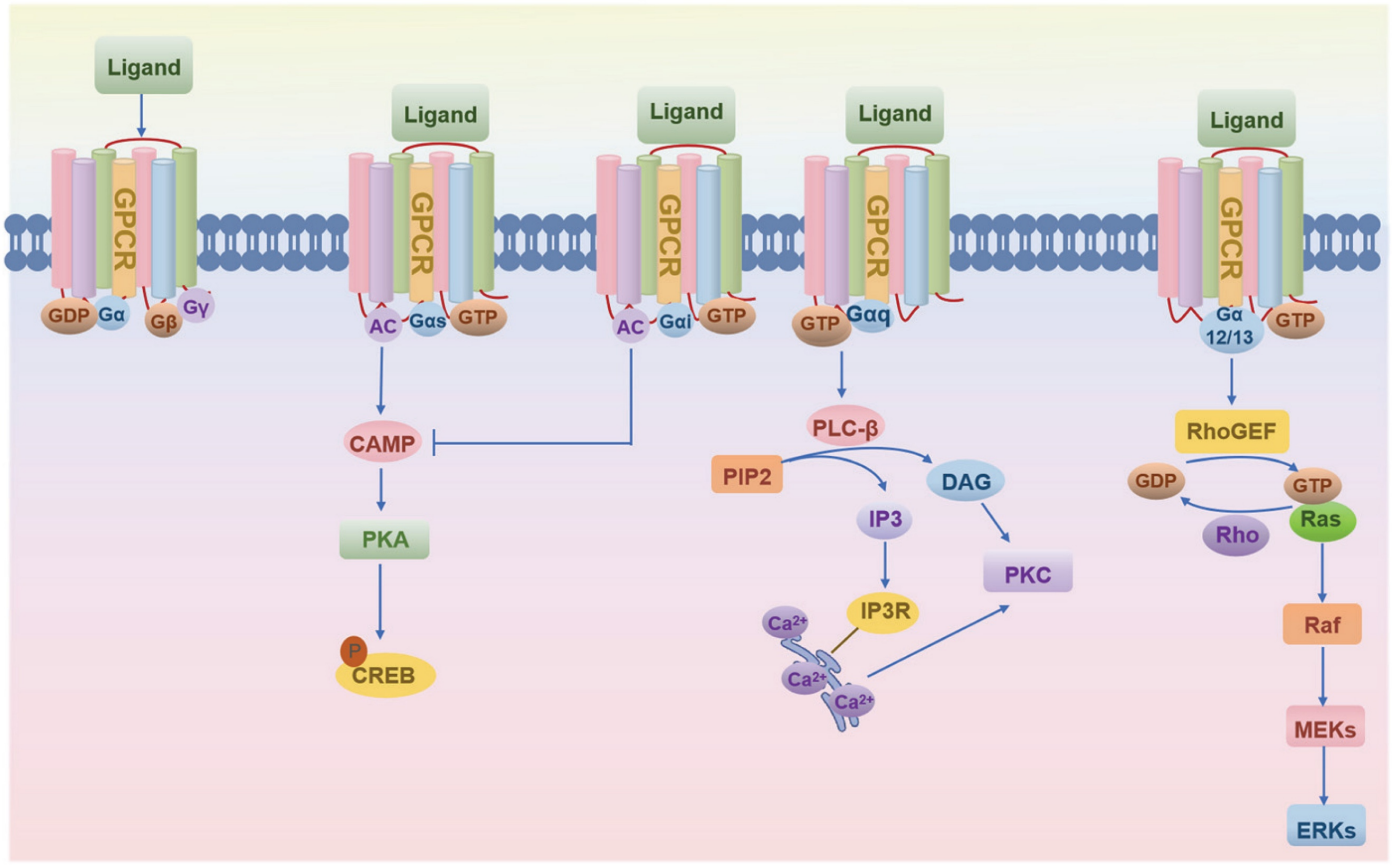
The schematic of GPCR-ligand binding and downstream G-protein signaling.

Since 1993, the skeletal research field has witnessed the identification of over 56 pivotal GPCRs as critical regulators of bone cell physiology (Fig. 2). Activation of these GPCRs by external signals triggers a series of biochemical reactions through interactions with distinct G proteins or arrestins, thereby regulating diverse physiological processes (Fig. 1 and Fig. 3) 4, 9. GPCRs mediate rapid signal transmission through G-protein-dependent pathways. The signaling cascade of GPCRs comprises four key stages: ligand-receptor binding, G protein activation, downstream pathway initiation, and cell-specific functional regulation 10, 11. GPCR signaling is initiated by extracellular ligands, including: hormones (e.g., PTH), ions (e.g., Calcium ions, Ca²⁺), lipid molecules (e.g., Lysophosphatidic acid, LPA; Sphingosine-1-phosphate, S1P), and chemokines (e.g., Stromal cell-derived factor-1, SDF-1) 10, 11. Ligand binding induces conformational changes in GPCRs, triggering G protein activation. GPCRs couple to heterotrimeric G proteins composed of α, β, and γ subunits 10, 11. At inactive state, Gα binds GDP and associates with Gβγ 10, 11. At activation process, ligand binding catalyzes GDP-to-GTP exchange on Gα. Then GTP-bound Gα dissociates from Gβγ, forming Gα-GTP and free Gβγ. After that both subunits activate downstream effectors, initiating distinct pathways. Functional specialization of G protein subtypes mainly includes Gs-type (e.g., Gαs) activates Adenylyl cyclase (AC), increasing cAMP. On the contrary, Gi inhibits the formation of cAMP catalyzed by AC. Gq-type (e.g., Gαq) activates Phospholipase C (PLC), hydrolyzing Phosphatidylinositol 4,5-bisphosphate (PIP2) into Diacylglycerol (DAG) and Inositol triphosphate (IP3). Gi-type (e.g., Gαi) inhibits AC, reducing cAMP. Gβγ complex independently activates pathways like Ras-MAPK. G protein subunits regulate cellular behavior via three core pathways, including cAMP- protein kinase A (PKA)-CREB pathway (Gs-dominant), PLC-protein kinase C (PKC)/Ca²⁺ pathway (Gq-dominant), and Ras-MAPK Pathway (Gβγ- or Gα12/13-dominant) (Fig. 3) 3, 10, 11. This pathway drives immediate physiological responses (e.g., metabolic regulation) and terminates signals via GTP hydrolysis and β-arrestin-mediated desensitization, enabling precise spatiotemporal control. Besides, GPCRs mediate prolonged signaling regulation through G-protein-independent pathways. Receptor phosphorylation recruits β-arrestin, forming complexes that activate MAPKs (e.g., ERK, JNK), Src family kinases, or NF-κB to regulate cell proliferation, stress responses, and gene expression. Signals persist longer in this pathway and may extend to perinuclear regions via endosomal trafficking, influencing nuclear functions. Biased ligands selectively targeting this pathway offer novel therapeutic opportunities for disease 4, 9. The diversity of GPCR signaling pathways also lays a good foundation for analyzing the function of GPCR in bone homeostasis, including the effect of Gαs/Gαi mediated on the function of osteoblasts/osteoclasts, etc.

The dawn of the 21st century ushered in a structural biology revolution propelled by advancements in cryo-electron microscopy (cryo-EM) and X-ray crystallography, which fundamentally reshaped GPCR research paradigms. In bone biology, this structural elucidation provided unprecedented mechanistic insights into how GPCRs integrate skeletal signals - from hormonal cues (e.g., PTH) to mechanical stimuli to modulate osteoblastogenesis, osteoclastogenesis, and chondrogenesis. The post-2000 era saw exponential acceleration in GPCR research velocity (Fig. 2). This convergent evolution of structural biology, pharmacology, and skeletal medicine positions GPCRs as central nodes in the quest for precision osteotherapeutics.

## GPCRs in MSCs

MSCs, known for their self-renewal capability and multilineage differentiation potential, are key players in tissue regeneration and repair, particularly in the bone marrow niche 12. These cells express a wide array of GPCRs that respond to a multitude of ligands, including hormones, neurotransmitters, growth factors, and small molecules (Table 1). The interaction between these ligands and their cognate GPCRs orchestrates a complex signaling network that fine-tunes the behavior of MSCs, thereby regulating bone homeostasis and repair.

**Figure 4:**
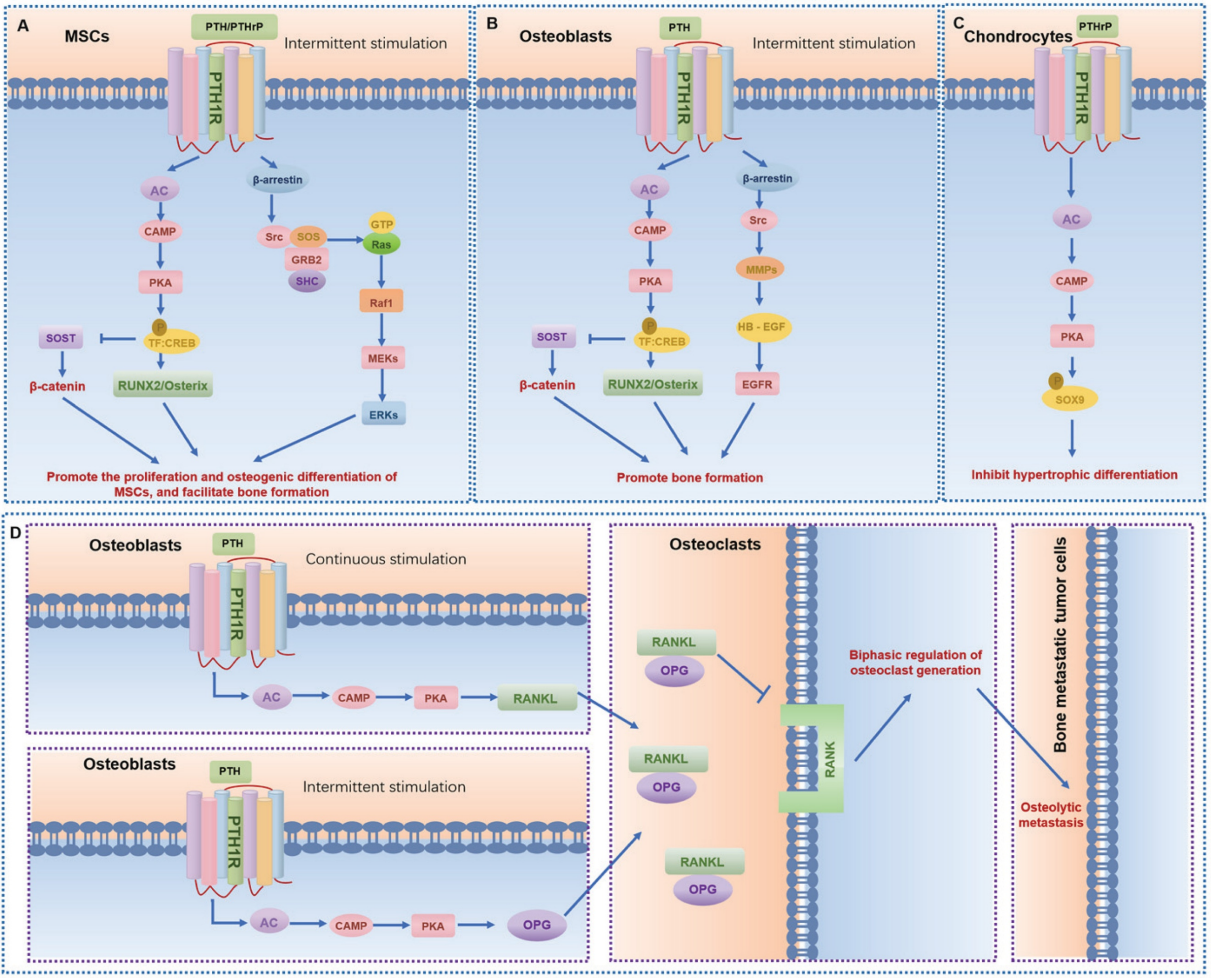
** The signaling pathways and functions of PTH1R in different bone-related cells. (A-C)** The main signaling pathways of PTH1R in mesenchymal stem cells (MSCs) (A), osteoblasts (B), and chondrocytes (C), respectively. **(D)** Osteoclasts are indirectly regulated by PTH1R in osteoblasts, leading to osteolytic metastasis and regulating the dynamics of other bone-related cells.

**Figure 5:**
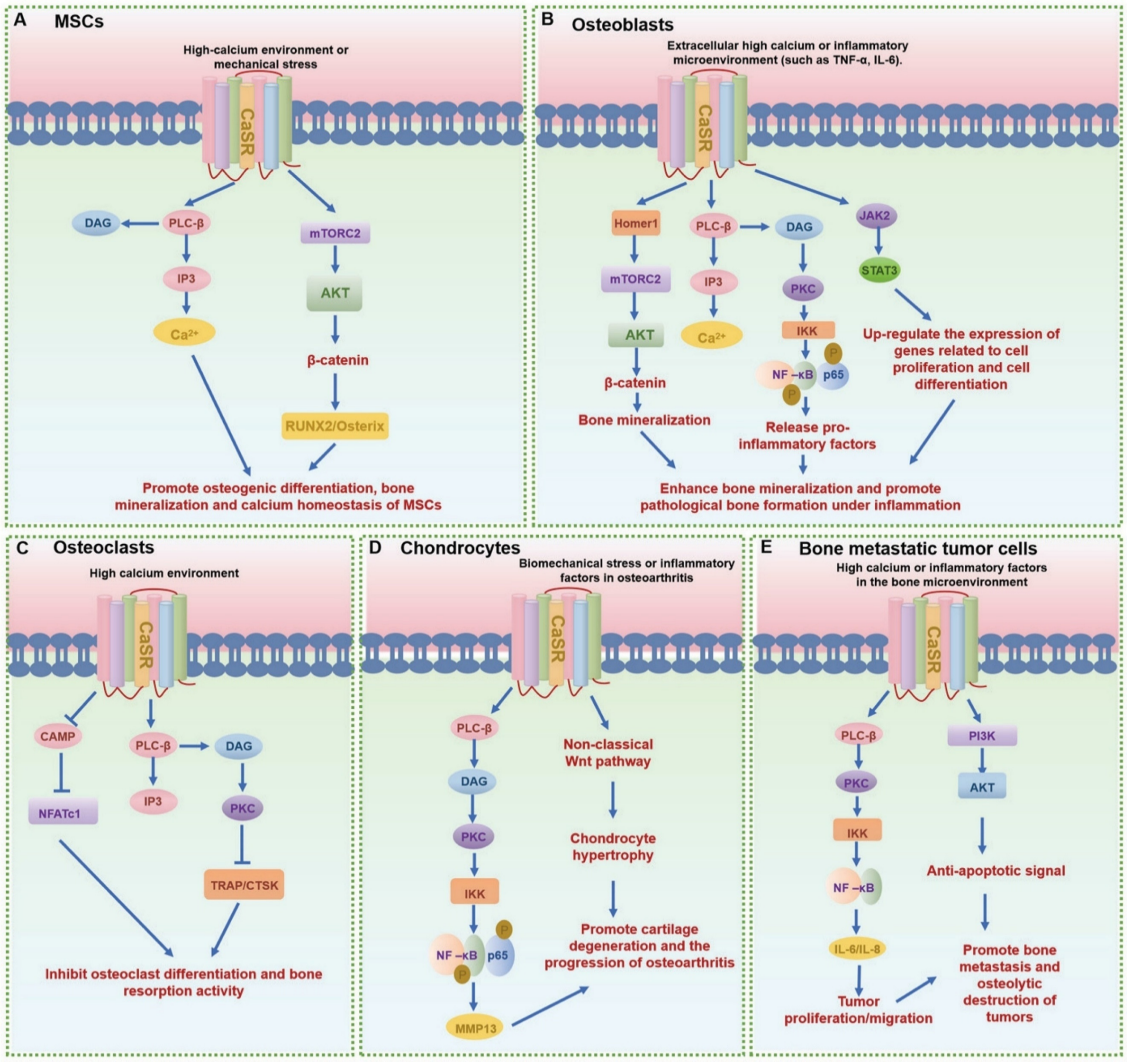
** The signaling pathways and functions of CaSR in different bone-related cells. (A-E)** The main signaling pathways of CaSR in mesenchymal stem cells (MSCs) (A), osteoblasts (B), osteoclasts (C), chondrocytes (D) and other bone-related cells (E), respectively.

GPCRs regulate MSCs differentiation and mineralization, with specific pathways promoting osteogenic differentiation (Table 1). Leucine-rich repeat containing G protein-coupled receptor 5 (LGR5), also known as G protein-coupled receptor 49 (GPR49), a vital member of the rhodopsin family of GPCRs and a Wnt target gene, plays a significant role in osteogenic differentiation 13. Lgr5 knockdown suppresses osteogenic differentiation via dysregulation of Wnt/ERK signaling and impaired mitochondrial dynamics, which are critical for MSC lineage commitment. Conversely, overexpression of Lgr5 accelerates fracture healing through enhanced osteogenesis and angiogenesis 14. Another crucial GPCR subset in MSCs is the Parathyroid hormone receptor 1 (PTH1R), a member of the class B GPCR family. PTH1R regulates MSCs functions through multiple signaling cascades: upon ligand binding (PTH or PTHrP), it activates Gαs protein to stimulate adenylate cyclase (AC), generating cAMP that activates protein kinase A (PKA) to upregulate osteogenic genes (e.g., Runx2, Osterix) and drive osteoblast differentiation; concurrently, PTH-PTH1R interaction recruits β-arrestin, forming a signaling complex that activates extracellular signal-regulated kinase 1/2 (ERK1/2), which promotes MSC proliferation and integrates survival signals through the MAPK pathway, collectively modulating skeletal homeostasis and cellular fate decisions (Fig. 4A) 6, 15. Moreover, CaSR, a class C GPCR, is vital for calcium homeostasis and bone turnover (Fig. 5A) 6, 15. Knockdown of CaSR (using shRNA-CaSR) decreases the bone formation potential of MSCs. CaSR signaling counteracts PTH1R signaling by downregulating PTH1R via inhibition of PTHrP expression 15. G protein-coupled receptor family C group 6 member A (GPRC6A), another GPCR, has been associated with human spine bone mineral density (BMD) 16. GPRC6A knockout mice exhibit lower BMD and a suppressed response to extracellular calcium-stimulated ERK activation, leading to attenuated osteogenic marker gene expression and mineralization in bone marrow mesenchymal stem cells (BMSCs) 16.

Adenosine receptors also play crucial roles in regulating MSC function. Adenosine A1 receptor (A1R) promotes osteogenic differentiation of human dental pulp stem cells (DPSCs) via Wnt signaling 17. Adenosine A2A receptor (A2AR) increases the proliferation and differentiation of MSCs from mouse bone marrow 18. The deletion of Adenosine receptor A2b (ADORA2B/A2BAR) results in lower BMD in mice, with decreased expression of osteoblast differentiation genes and fewer mineralized nodules in BMSCs 19. Prostaglandin E receptors (Prostaglandin E receptor 1, EP1/PTGER1; Prostaglandin E receptor 2, EP2/PTGER2; and Prostaglandin E receptor 4, EP4/PTGER4) also influence MSC function. Deletion of mice in enhanced fracture healing, stronger cortical bones, and higher trabecular bone volume 20, 21. This is due to inactivation of Hif1α, leading to increased oxygen consumption rate and promotion of osteogenic differentiation and bone formation 20. GPCR signaling also plays a regulatory role in the adipogenic differentiation of MSCs. Adrenoceptor β2 (ADRB2, also known as β-AR) agonists, on the other hand, suppress MSC mineralization in a dose- and time-dependent manner and inhibit adipogenesis and osteogenesis via the cAMP/PKA signaling pathway. ADRB2 antagonists have the opposite effect, increasing calcium mineralization and adipogenesis in MSCs 22, 23.

GPCRs assume a pivotal role in influencing over various cellular functions of MSCs, including proliferation and migration. Inhibition of CaSR disturbs the proliferation and migration of human BM-MSCs 24. CaSR in MSCs utilizes the G protein-dependent Gq/11-PLC-IP3 pathway: upon binding to Gq/11 proteins, CaSR activates PLC, catalyzing the hydrolysis of PIP2 into inositol IP3 and DAG. IP3 induces Ca²⁺ release from the endoplasmic reticulum, elevating cytoplasmic Ca²⁺ levels to activate calcium-dependent pathways (e.g., CaMK and PKC), thereby regulating MSCs proliferation and differentiation (Fig. 5A). In osteoarthritis, MSCs can serve as a substitute for chondrocytes and support cartilage regeneration. Norepinephrine, acting via ADRB2, suppresses the proliferation of BMSCs, thereby reducing their regenerative capacity. This suggests that targeting ADRB2 signaling may provide a novel therapeutic option for osteoarthritis 25. Lysophosphatidic acid (LPA) protects human umbilical cord MSCs (UC-MSCs) from LPS-induced apoptosis by inhibiting caspase-3 activation through Lysophosphatidic acid receptor 1 (LPAR1, also known as Endothelial differentiation gene 2, EDG2; or G protein-coupled receptor 26, GPCR26) coupled with a G protein. LPAR1 regulates LPA-induced proliferation of UC-MSCs, enhancing their survival without affecting differentiation 26. EP2 enhances the migration of MSCs by activating FAK and ERK1/2 pathways, without affecting osteogenic differentiation 27.

MSCs have immune regulatory functions, and GPCR signaling can affect the immune regulatory properties of MSCs. MSCs co-cultured with rheumatoid arthritis CD4^+^ T cells show that EP2/EP4-stressed MSCs have a better inhibitory effect on rheumatoid arthritis (RA) T cells by releasing PGE2, indicating that induction of EP2/EP4 stress can enhance the immunosuppressive effect of MSCs 28. GPCRs responsive to adrenergic ligands, such as β-adrenergic receptors, play significant roles in regulating MSC function under stress conditions. Activation of these receptors by catecholamines like adrenaline and noradrenaline stimulates cAMP production, leading to increased MSC migration and proliferation in response to injury or inflammation. However, chronic adrenergic stimulation can negatively impact osteogenic differentiation, highlighting the GPCR signaling in MSCs.

GPCRs are not only targets for exogenous ligands but also sensors of the mechanical microenvironment. Primary cilia, an important organelle in bone mechanobiology and mechanical transduction, harbor GPCRs that respond to mechanical stimuli. G protein-coupled receptor 161 (GPR161), a mechanically responsive orphan GPCR located in the cilium, is crucial for fluid shear-induced cAMP signaling in MSCs 29. The absence of GPR161 inhibits mechanical transduction, leading to decreased expression of osteogenic marker genes downstream 29. This mechanosensitive is pivotal in bone adaptation to mechanical loading and in the pathogenesis of bone diseases such as osteoporosis.

In summary, 12 GPCRs orchestrate MSC functions, including differentiation, proliferation, migration, and immune regulation (Table 1). Key receptors include LGR5, which promotes osteogenesis via Wnt/ERK signaling and mitochondrial dynamics, accelerating fracture healing. PTH1R stimulates osteoblastogenesis through cAMP/PKA-mediated Runx2 and osteocalcin expression, enhancing bone formation (Fig. 4A). CaSR maintains calcium homeostasis (Fig. 5A). Adenosine receptors (A1R, A2AR, A2BAR) modulate osteogenic differentiation and proliferation. Prostaglandin E receptors (EP2/EP4) enhance MSCs migration and fracture repair via FAK/ERK pathways, while suppressing adipogenesis. ADRB2 regulate cAMP/PKA signaling, impacting MSC mineralization. Mechanically responsive GPCR like GPR161 mediates osteogenic responses to fluid shear stress and matrix stiffness. Understanding the intricate GPCR signaling networks in MSCs will pave the way for the development of novel therapeutic strategies to treat bone diseases, enhance bone regeneration, and improve the efficacy of MSC-based therapies.

## GPCRs in osteoblast and osteocyte

In bone tissue, osteoblasts and osteocytes collaborate to maintain skeletal homeostasis. Osteoblasts, responsible for bone formation, synthesize and mineralize the organic matrix to generate new bone, while osteocytes, embedded within the mineralized matrix, orchestrate bone remodeling through mechanotransduction and intercellular signaling. GPCRs emerge as critical regulators of these processes, mediating diverse signaling pathways that govern osteoblast differentiation, osteocyte function, and overall bone metabolism (Table 2).

GPCRs exert bidirectional control over osteoblast activity, influencing both pro-osteogenic and anti-osteogenic pathways. Pro-osteogenic signaling pathways include the activation of the Wnt/β-catenin pathway by receptors such as LGR5 and Leucine-rich repeat-containing G protein-coupled receptor 4 (LGR4, also known as G protein-coupled receptor 48, GPR48), which promote osteoblast differentiation 30 31. LGR4 deficiency, for instance, delays osteoblast mineralization during embryonic bone development, yet does not impair chondrocyte maturation, highlighting its specificity for osteogenic signaling 31. CaSR and PTH1R are pivotal for calcium homeostasis, with CaSR alleles associating with BMD and osteoporosis risk, and PTH1R agonists (e.g., teriparatide) serving as therapeutic agents for osteoporosis by enhancing bone formation (Fig. 4B,D and Fig. 5B) 32-35. Additionally, LGR4 regulates osteoblast differentiation via the cAMP-PKA-ATF4 pathway, underscoring its role in early bone development 31. G protein-coupled receptor 39 (GPR39), another critical GPCR, is involved in bone matrix deposition, with Gpr39^-/-^ mice exhibiting disordered matrix deposition characterized by abnormally low collagen and high mineral contents in osteoblasts 36.

GPCRs also mediate anti-osteogenic and modulatory signaling. Adrenergic receptors exemplify this duality: Adrenoceptor β1 (ADRB1) agonists mitigate disuse-induced bone loss by reducing osteocyte apoptosis 37, while ADRB2 deficiency increases bone mass, and ADRB2 agonists stimulate RANKL expression in osteoblasts, promoting osteoclastogenesis and bone resorption 38-40. ADRA1A (α1-adrenergic receptor, α1-AR) signaling upregulates the transcriptional repressor Nfil3, inhibiting BMP4 expression and establishing a circadian regulatory loop 41, and ADRA2A (α2A-adrenergic receptors, α2A-AR) polymorphisms correlate with altered bone resorption markers (e.g., CTX, Cathepsin K) and osteoporosis risk 42. The overexpression of GRK2, a kinase that terminates GPCR signaling, suppresses osteoblast numbers and bone formation by attenuating Wnt/β-catenin activity, leading to low bone turnover 43, 44. GABAB receptor (GABABR) deficiency increases ALP activity and BMP2/Osterix expression in osteoblasts, disrupting osteoclastogenesis via RANKL downregulation 45. GPR161, a cilium-localized GPCR, is essential for intramembranous bone formation, with Gpr161 knockout mice lacking forelimbs due to hyperactive Sonic Hedgehog (Shh) signaling and blocked osteoblastogenesis 29, 46. GPRC6A deficiency suppresses calvarial-derived osteoblast differentiation and Alkaline phosphatase (ALP) activity, with siRNA-mediated knockdown of Gprc6a in MC3T3-E1 osteoblasts restraining extracellular calcium-stimulated ERK activities 16.

The roles of GPCRs in bone metabolism are context-dependent. LPAR1 deficiency impairs bone mineralization and cortical thickness, accompanied by osteocyte apoptosis and lacunar defects 47. Metabotropic glutamate receptor 1 (GRM1, also known as mGluR1 or mGlu1) knockout mice exhibit premature growth plate fusion and osteoblast dysfunction, linking glutamate signaling to skeletal maturation 48. EP2A enhances osteoblastic differentiation and mineralization 49, while adenosine receptors, including A1R and ADORA2B, play roles in osteoblast and adipocyte lineage determination 50, 51, with A2AR agonists promoting new bone formation by increasing osteoblast activity and reducing osteoclast activity 50, 52.

Osteocytes, the most abundant bone cells, utilize GPCRs to maintain mechanical integrity and coordinate remodeling. ADRA2A (α2A-adrenergic receptors, α2A-AR) in osteoblasts and lining cells mediates neuroendocrine inputs, with single nucleotide polymorphisms (SNP rs553668 and rs1800544) affecting bone resorption markers 42. CaSR signaling in osteoblasts is modulated by inflammatory cytokines (e.g., NF-κB/JAK-STAT3), influencing pathological bone formation in ankylosing spondylitis (Fig. 5B) 34. Homer1 mediates CaSR signaling via mTORC2 in osteoblasts to enhance AKT-dependent β-catenin stabilization, while systemic inhibition of CaSR represses pathological new bone formation in animal models of ankylosing spondylitis 33. In the inflammatory immune responses of osteoblasts, pulsed electromagnetic fields promote the anti-inflammatory effect of A2A and Adenosine A3 receptor (A3AR) in human hFOB 1.19 osteoblasts 53.

Genetic and therapeutic insights further highlight the importance of GPCRs in bone biology. An intergenic susceptibility SNP rs4683184, influences transcription factor RUNX2 binding and mediates long-range chromatin interactions with X-C chemokine receptor 1 (XCR1) 54. XCR1, also named as G protein-coupled receptor 5 (GPR5), promotes osteoblast differentiation, and the bone-targeting adeno-associated virus targeting Xcr1 enhances bone formation in osteoporotic mice 54. Therapeutically, PTH1R agonists (teriparatide, abaloparatide) are first-line treatments for osteoporosis, while Cannabinoid receptor 2 (CNR2/CB2) agonists show promise in preventing ovariectomy-induced bone loss by stimulating osteogenesis 55, 56.

In conclusion, 21 GPCRs form a complex regulatory network in bone biology, integrating hormonal, mechanical, and metabolic cues to fine-tune osteoblast and osteocyte activity (Table 2). LGR5, PTH1R, CNR2, and EP2A play important roles in promoting bone formation. On the contrary, GPRC6A, and ADRB2 agonists mainly inhibit bone formation. In terms of bone resorption regulation, ADRB2 and A2AR have become key driving factors. CaSR and A2AR/A3AR play crucial roles in inflammation and immune regulation. LGR4, GPR39 and A1R also affect development and metabolic regulation. Understanding the functional roles and signaling cascades of these receptors will pave the way for the development of novel therapeutic strategies to treat bone diseases and enhance bone regeneration.

## GPCRs in macrophage and osteoclast

GPCRs play pivotal roles in orchestrating the migration, differentiation, and activation of macrophages and osteoclasts, thereby profoundly influencing bone mass, microstructure, and strength (Table 3). These receptors mediate complex signaling networks that either promote or inhibit osteoclastogenesis and macrophage polarization, with implications for skeletal homeostasis and disease.

As a rhodopsin-family GPCR, Thyroid-stimulating hormone receptor (TSHR/LGR3) suppresses osteoclast activity by inhibiting RANKL-induced differentiation, as evidenced by increased bone resorption in Tshr^-/-^ mice and reduced TRAP-positive osteoclasts following TSH treatment 57. EBV-induced G protein-coupled receptor 2 (EBI2, also known as GPR183) and its ligand 7α,25-dihydroxycholesterol (7α,25-OHC), secreted by osteoblasts, guide osteoclast precursor (OCP) migration to bone surfaces, with EBI2 deficiency enhancing bone mass by disrupting OCP homing 58. G protein-coupled receptor 55 (GPR55) modulates osteoclastogenesis by attenuating RANKL-stimulated transcription of osteoclast markers, while its inhibition via peptide P1 blocks osteoclast maturation 59. Similarly, Coagulation factor II thrombin receptor (F2r) restrains osteoclast formation and function by attenuating RANKL-induced signaling through the Akt and NF-κB pathways 60.

In addition, quercetin, acting through G protein-coupled receptor 30 (GPR30) rather than nuclear estrogen receptors, inhibits osteoclastogenesis, highlighting crosstalk between cytokine and GPCR pathways 61. LGR4 competes with RANK for RANKL binding, initiating cAMP-PKA-CREB signaling that upregulates Atf4 in osteoblasts and counteracts RANK-mediated osteoclast activation 31. Postnatal Lgr4 deficiency increases osteoclast activity, underscoring its role in fine-tuning bone remodeling 62, 63. G protein-coupled receptor G2A (GPR132) plays a role in macrophage migration and polarization during inflammation. In G2A-deficient mice, there is reduced M1-like macrophage infiltration at the site of inflammation and a shift towards M2-like polarization, highlighting the importance of GPCRs in regulating macrophage function during immune responses 64. G protein-coupled receptor 120 (GPR120, also known as Free fatty acid receptor 4, FFAR4) activation by TUG-891 inhibits osteoclast formation and resorption in RAW264.7 macrophages by reducing ROS and upregulating antioxidant proteins 65. Recently, the study reveals that G protein-coupled receptor 54 (GPR54), activated by Kisspeptin-10 (Kp-10), recruits Dusp18 phosphatase to dephosphorylate Src at Tyr416. Knockout of Kiss1, Gpr54, or Dusp18 in mice results in osteoclast hyperactivation and bone loss 66. Kp-10 treatment suppresses osteoclast activity and bone loss *in vivo* 66. Thus, the Kp-10/Gpr54 pathway represents a potential therapeutic target for bone resorption via Dusp18-mediated Src dephosphorylation 66.

CaSR, expressed in mature osteoclasts, inhibits bone resorption in response to high extracellular Ca²⁺ or agonists, with osteoblast-specific CaSR knockout upregulating RANKL and increasing osteoclast activity (Fig. 5C) 67-71. GABABR in osteoblasts suppresses cAMP formation, ALP activity, and osteogenic genes (e.g., BMP2, Osterix), thereby reducing osteoblastogenesis and indirectly modulating osteoclastogenesis via RANKL 45. Undercarboxylated osteocalcin (ucOCN) inhibits early osteoclast differentiation through GPRC6A 72, while Taste 1 receptor member 3 (Tas1R3) deficiency enhances cortical bone mass by uncoupling bone remodeling and reducing osteoclast function 73.

Adenosine receptors exhibit opposing effects. A1R promotes osteoclastogenesis, whereas A2AR inhibits differentiation and function 74. A2AR agonists enhance bone regeneration by increasing osteoblasts and decreasing osteoclasts in skull defects, while Adora2b deficiency reduces bone mass and trabecular number 52, 75. ADRB2 signaling in periodontal ligament cells (PDLCs) stimulates osteoclastogenesis and accelerates orthodontic tooth movement via RANK-L upregulation, with noradrenaline and selective agonists enhancing osteoclast multinuclearity without directly affecting osteoblasts 76, 77.

Moreover, cannabinoid receptors also regulate bone turnover. Cannabinoid receptor 1 (CNR1) antagonism increases bone mass by promoting osteoclast apoptosis 78, while combined Cnr1/Cnr2 deficiency protects against age-related and ovariectomy-induced bone loss despite reduced osteoblast function 78, 79. In a mouse model of diet-induced obesity, treatment with the Cannabinoid receptor 1 (CB1) antagonist AM251 resulted in weight loss and reduced inflammation 80. CB2 (CNR2) agonists stimulate osteoclastogenesis, with CB2^-/-^ mice exhibiting blunted ovariectomy-induced bone loss, suggesting therapeutic potential for CB2 antagonists 81. Additionally, Dopamine receptor D2 (DRD2) suppresses M1 macrophage polarization and NF-κB/NLRP3 inflammasome activation 82, while LPAR1/EDG2/GPCR26 deficiency impairs osteoclastogenesis and prevents ovariectomy-induced bone loss 83. Recently, the study found that G protein-coupled receptor 125 (GPR125) is highly expressed in osteoclasts and positively regulates their differentiation and activation 84. Additionally, GPR125 knockdown reduced the expression of phosphorylated MAPK (p-ERK and p-p38) and AKT-NF-κB (p-AKT and p-IKBα) signaling pathway proteins in response to RANKL stimulation 84.

PGE2 receptors exhibit context-dependent roles. EP4 downregulation in osteoclasts prevents PGE2-mediated inhibition of bone resorption, yet EP4 on osteoblasts is critical for osteoclast formation induced by inflammatory cytokines 85-87. Besides, Histamine H4 receptor (H4R), also known as GPCR105, blockade reduces RANKL expression and osteoclastogenesis in rheumatoid arthritis, where synovial histamine levels correlate with disease severity 88.

Collectively, these evidences emphasize the crucial role of 23 GPCRs in regulating macrophage and osteoclast function (Table 3). A1R, ADRB2, LPAR1, GPR55, H4R promote osteoclastogenesis, while GPR120, GPR54, A2AR, CNR1, GPRC6A inhibit osteoclastogenesis. The regulation of bone resorption is regulated by CaSR, PTH1R, F2r, and GIT1. Immune and inflammatory regulation are also regulated by GPR132, EP4, and H4R. In terms of metabolism and nutrient sensing, the role of TASR3 and GPR120 cannot be ignored. Their diverse roles in osteoclastogenesis, macrophage polarization, and osteoblast-osteoclast crosstalk underscore their therapeutic potential in osteoporosis, inflammatory arthritis, and metabolic bone diseases. Targeting these receptors offers innovative strategies to modulate bone resorption and formation, with implications for regenerative medicine and anti-resorptive therapies.

## GPCRs in chondrocyte

GPCRs serve as master regulators of chondrocyte biology, orchestrating responses to growth factors, cytokines, mechanical cues, and inflammatory mediators. These receptors govern critical processes such as chondrocyte proliferation, differentiation, extracellular matrix (ECM) synthesis, and survival, thereby maintaining cartilage integrity and modulating the pathogenesis of osteoarthritis (Table 4). Dysregulation of GPCR signaling contributes to cartilage degradation, synovial inflammation, and subchondral bone remodeling, highlighting their therapeutic potential in osteoarthritis management.

Chemokine receptors play crucial roles in regulating chondrocyte biology and osteoarthritis pathogenesis. During endochondral ossification, C-X-C motif chemokine receptor 4 (CXCR4) is highly expressed in hypertrophic chondrocytes at the chondro-osseous junction 89. Here, CXCR4 binds stromal cell-derived factor 1 (SDF-1) secreted by adjacent osteoblasts and marrow stromal cells, forming a positive feedback loop with RUNX2 to amplify hypertrophic differentiation and type X collagen expression 89. In rabbit models, SDF-1 infiltration accelerates growth plate hypertrophy and premature physeal closure, underscoring CXCR4's role in skeletal maturation 89. Conversely, C-X-C motif chemokine receptor 2 (CXCR2) deficiency exacerbates osteoarthritis pathology by reducing ECM production (e.g., aggrecan, type II collagen) and increasing chondrocyte apoptosis via attenuated AKT signaling 90. C-X-C motif chemokine receptor 3 (CXCR3) expression was significantly elevated in osteoarthritis patients 91. By using siRNA to downregulate CXCR3 in chondrocyte models induced by IL-1β and sodium nitroprusside, it was found that CXCR3 reduction had no effect on IL-1β-induced chondrocyte apoptosis but significantly decreased nitrate levels 91. However, it markedly reduced nitrate levels and alleviated sodium nitroprusside-induced chondrocyte apoptosis 91. The UPR pathway factors CHOP and GRP78 were involved in this process, suggesting that CXCR3 modulates chondrocyte apoptosis via the ER stress signaling pathway 91.

The C-C chemokine receptor family also plays pivotal roles in osteoarthritis pathogenesis. C-C motif chemokine receptor 5 (CCR5) ablation protects against cartilage degeneration in post-traumatic osteoarthritis models, independent of synovial or bone changes, suggesting a cartilage-specific protective role 92. Interestingly, another study found that mice lacking C-C motif chemokine ligand 2 (CCL2) or C-C motif chemokine receptor 2 (CCR2), but not CCL5 or CCR5, were protected against osteoarthritis with reduced monocyte/macrophage recruitment 93. Elevated levels of CCR2 ligands were found in synovial fluids from osteoarthritis patients 93. CCR2^+^ macrophages were abundant in human osteoarthritis synovium and associated with cartilage erosion 93. Blocking CCL2/CCR2 signaling significantly reduced macrophage accumulation, synovitis, and cartilage damage in mouse osteoarthritis, suggesting that selective targeting of the CCL2/CCR2 system may be a very promising therapeutic approach for osteoarthritis 93.

Emerging evidence reveals additional GPCRs as modulators of chondrocyte stress responses. Proteinase-activated receptor 2 (PAR2) emerges as a key mediator of osteoarthritis inflammation and structural damage. Studies employing PAR2 knockout mouse models have demonstrated a reduction in knee swelling and cartilage damage severity compared to wild-type mice, thereby highlighting the involvement of PAR2 in structural alterations within osteoarthritic joints 94. Specifically, the PAR2 antagonist AZ3451 has been shown to alleviate inflammation, cartilage degradation, and cellular senescence in chondrocytes, while promoting autophagy to decrease apoptosis 95. Notably, PAR2 deficiency in mice also leads to decreased osteophyte formation and absence of osteosclerosis, indicating its role in bone changes associated with osteoarthritis 96. These findings collectively underscore the therapeutic potential of PAR2 antagonists in treating osteoarthritis by addressing multiple disease facets, including pain perception and bone pathology.

Melanocortin receptors MC1R/MC3R (Melanocortin 1 receptor/ Melanocortin 3 receptor) exert chondroprotective effects by suppressing inflammatory cytokines and matrix-degrading enzymes while promoting anti-inflammatory mediators 97. H4R expression correlates with type X collagen (COLX)-positive hypertrophic chondrocytes, suggesting involvement in terminal differentiation 98. Prostaglandin E2 (PGE2) receptors exhibit subtype-specific functions 99. EP1 negatively regulates bone formation, while combined EP2/EP4 stimulation is required for type II collagen upregulation 21. EP2 agonists suppress MMP-13 expression via cAMP-PKA signaling, demonstrating anti-catabolic effects without compromising cell viability 100.

Multiple GPCRs exhibit protective profiles in cartilage biology. Opioid receptor kappa 1 (OPRK1/KOR) signaling enhances anabolic activity through cAMP/CREB pathways 101, while G protein-coupled receptor 40 (GPR40) agonists reduce metalloproteinase expression and NF-κB activation 102. GPR43 activation by butyrate mitigates IL-1β-induced inflammation 103, and GPBAR1 activation protects against senescence 104. GPR4 promotes osteoarthritis progression through NF-κB/MAPK signaling, making its inhibition a therapeutic target 105. GPR120 agonists preserve matrix components via SOX9-mediated pathways 106, and GPR84 deficiency exacerbates cartilage catabolism through impaired NF-κB regulation 107.

Adrenergic signaling demonstrates contextual regulation 108. β-AR (also known as ADRB2) activation inhibits chondrogenic differentiation through ERK1/2-mediated AP-1 signaling 109, 110, while α2A-AR stimulation drives matrix degradation via ERK1/2/PKA pathways 111, 112. Sympathetic nerve-derived norepinephrine exhibits dose-dependent effects—low concentrations promote proliferation/apoptosis through α1-AR 113, while high concentrations maintain phenotypic stability via β-AR 113. Adenosine receptors modulate cartilage integrity through distinct mechanisms: A3AR agonists suppress RUNX2/CaMKII to prevent matrix degradation 108, while A2AR stimulation enhances autophagy via FOXO activation 114. Besides, local injection of adrenoreceptor antagonists or agonists showed that α2A-AR activation in chondrocytes leads to cartilages degeneration and subchondral bone loss by suppressing aggrecan expression and stimulating MMP-3, MMP-13, and RANKL production via ERK1/2 and PKA pathways 111. Inhibiting α2A-AR attenuated degenerative changes, while activating it intensified them 111. Thus, α2A-adrenergic signal activation in chondrocytes accelerates temporomandibular joint degenerative remodeling 111. Moreover, Bai's team developed a cartilaginous organoid system from hEPSCs with dual reporters to monitor chondrogenesis and hypertrophy, identifying α-adrenergic receptor antagonists (e.g., phentolamine) as enhancers of chondrogenesis and suppressors of hypertrophy, while α2-AR agonists induced detrimental hypertrophy 112. Mechanistically, α2-AR signaling drives hypertrophic degeneration via cGMP-dependent SLPI production, and targeting this pathway, including SLPI inhibition, shows therapeutic potential for regenerating hyaline cartilage and repairing defects without fibrosis 112.

Angiotensin receptors demonstrate balanced regulation, with Angiotensin II receptor type 1 (AT1R) inhibition promoting proliferation and Angiotensin II receptor type 2 (AT2R) activation mitigating apoptosis under oxidative stress 115. Cannabinoid receptors CB1/CB2 regulate skeletal growth and osteoarthritis progression 116. CB1 deficiency causes femoral elongation defects, while CB2 activation protects against osteoarthritis through SIRT1 upregulation and senescence inhibition 117.

In the pathophysiology of cartilage and osteoarthritis, PTH1R mediates anabolic responses to intermittent PTH administration, promoting bone formation and subchondral bone integrity 118. After activation of PTH1R, it upregulates the level of cAMP, thereby activating PKA and regulating gene expression in chondrocytes (Fig. 4C). Moreover, PTH1R activation modulates the subchondral bone microenvironment by suppressing aberrant bone remodeling, reducing sensory nerve innervation and vascular invasion, thereby decreasing inflammatory mediators like PGE2 119. This process not only alleviates pain but also slows osteoarthritis progression through preservation of Nestin^+^ mesenchymal stem cell-driven bone remodeling 7. Conversely, CaSR, activated by abnormal biomechanical stimuli (e.g., fluid shear stress) in osteoarthritis, induces endoplasmic reticulum calcium overload, accelerating chondrocyte hypertrophy and matrix degradation 15, 24, 33. Abnormal fluid shear stress activates CaSR and promotes chondrocyte hypertrophy and the expression of stromal degradation enzymes (such as MMP-13) through the MAPK/NF-κB pathway (Fig. 5D). CaSR inhibition emerges as a therapeutic strategy to mitigate these pathological processes 120. Additionally, CaSR contributes to subchondral bone metabolic dysregulation, with its hyperactivation exacerbating osteoarthritis progression through aberrant bone remodeling. Collectively, PTH1R and CaSR represent opposing regulators in osteoarthritis: PTH1R exerts protective effects via bone-cartilage crosstalk, while CaSR drives pathological differentiation and matrix breakdown, highlighting their therapeutic potential as targets for osteoarthritis management.

Overall, these findings underscore the 31 GPCRs in regulating chondrocyte activity and differentiation, offering potential therapeutic targets for the treatment of bone and cartilage-related disorders such as fracture healing and osteoarthritis (Table 4). Such as promoting cartilage protection, MC1R/MC3R, GPR120, KOR, GPR40, A3AR, A2AR are indispensable. CCR2, GPR4, α2A-AR, and CaSR will accelerate the progression of osteoarthritis. EP2/EP4, CXCR2, and GPR43 mainly play a role in regulating ECM. PAR2, GPR43, A2AR, CB1/CB2 are related to inflammation regulation. CXCR4, H4R, and EP1 play important roles in the direction of chondrocyte hypertrophy and differentiation. This comprehensive GPCR network integrates diverse signals to maintain cartilage integrity. Targeting these receptors offers opportunities to modulate inflammation, matrix turnover, and cellular senescence—key pathological drivers in osteoarthritis.

## GPCRs in other cells

GPCRs also play pivotal roles in other bone-related cells, such as synovial fibroblasts, immune cells, adipocytes, muscle cells and tumor cells, exerting significant influences on a multitude of physiological and pathological processes 121-123. For instance, they impact tumor bone metastasis, a critical aspect in the progression of certain cancers where the interaction between GPCRs and bone cells can facilitate the metastatic spread of tumor cells to bone tissues (Table 5). Calcium, via overexpressed CaSR, promotes the migration and proliferation of bone-metastasizing renal cell carcinoma (RCC) cells by activating downstream pathways (Fig. 5D). Thus, CaSR could serve as a novel prognostic marker for RCC bone metastasis 122. A3AR, which shares similar biological properties with TMIGD3 (Transmembrane and immunoglobulin domain containing 3) isoform 1, also functions as a suppressor of osteosarcoma cell aggressiveness by inhibiting the PKA-Akt-NF-κB signaling axis 124.

Furthermore, GPCRs are involved in regulating insulin sensitivity, which is crucial for maintaining metabolic homeostasis and preventing conditions such as diabetes. In diabetic mouse models induced by streptozotocin or a high-fat diet, AR420626 elevated plasma insulin levels, increased skeletal muscle glycogen content, and improved glucose tolerance 123. Activation of G protein-coupled receptor 41 (GPR41) with AR420626 enhanced glucose uptake in muscle cells by boosting calcium signaling 123. These findings indicate that GPR41 is a promising target for diabetes treatment, as it can enhance insulin sensitivity and glucose regulation. Selectively knocking down CB1R in Kupffer cells improves glucose tolerance and insulin sensitivities in obese mice without influencing hepatic lipid contents or body weight. This effect is associated with a shift to an anti-inflammatory cytokine profile and enhanced insulin signaling, indicating that CB1R in Kupffer cells plays a crucial role in obesity-related hepatic insulin resistance through a pro-inflammatory mechanism 125. In diabetic mouse models, GPR41/FFAR3 activation by AR420626 improved glucose tolerance via enhanced calcium signaling, elevated insulin levels, and increased muscle glycogen content 123. These findings indicate that GPR41 is a promising target for diabetes treatment, as it can enhance insulin sensitivity and glucose regulation.

Additionally, they modulate immune responses and inflammatory pathways, thereby influencing the body's defense mechanisms and its ability to manage inflammation, which are particularly relevant in bone health and disease. Osteoimmunology is receiving increasing attention, as there exist numerous shared molecules between the immune system and the skeletal system, including members of the GPCR family. These GPCRs mediate bone-immune crosstalk, providing critical insights into bone diseases and immune disorders. Persistent inflammation in impaired joints leads to metabolic dysregulation in the synovial microenvironment, altering cell activity and contributing to rheumatoid arthritis pathogenesis. Recent research highlights the role of metabolite-sensing GPCRs in rheumatoid arthritis related inflammatory immune responses. Some GPCRs influence RA progression by modulating immune cell behavior 121. Additionally, a study identified a significant association between a SNP in the promoter region of the EDG2 gene, which encodes an LPA receptor, and knee osteoarthritis in two independent populations 126. The susceptibility allele of this SNP enhances transcriptional activities and DNA binding in synovial cells, leading to increased expression of inflammatory cytokines and matrix metalloproteases 126. These findings indicate that the LPA-EDG2 signal contributes to the pathogenesis of osteoarthritis through catabolic processes 126.

Moreover, GPCRs assume a pivotal function in the course of bone metastasis. G protein-coupled receptor class C group 5 member A (GPRC5A) knockout PC3 cells fail to establish bone metastasis in mice 127. The expression of GPRC5A correlates with bone metastasis and the Gleason score in prostate cancer patients, suggesting its potential as a therapeutic target and prognostic marker for advanced prostate cancer 127. LSSIG, a novel murine leukocyte-specific GPCR induced by STAT3 activation, has high homology to human GPR43. The expression of LSSIG is induced in M1 leukemia cells during differentiation to macrophages in a STAT3-dependent manner 128. Similarly, GPR43 expression is induced during the differentiation of HL-60 and U937 leukemia cells to monocytes 128. Both LSSIG and GPR43 are highly restricted to hematopoietic tissues and are induced by cytokine stimulation in bone marrow cells, monocytes, and neutrophils 128. These findings indicate that LSSIG and GPR43 may play vital roles in the differentiation and immune response of monocytes and granulocytes 128. In addition, CXCL12 promotes EMT-like changes and osteotropism in CXCR4 high/CXCL12 low neuroendocrine tumor (NET) cells via CXCR4. Silencing CXCR4 abrogates CXCL12-induced EMT, migration, and mesenchymal transcriptional patterns 129. The subcellular localization of CXCR4 may suggest unique functions, hinting at potential relevance for future *in vivo* studies 129. Notably, a CXCR4 agonist pepducin, a synthetic molecule that combines a peptide from CXCR4's intracellular loop with a lipid tether, has been found to mobilize bone marrow hematopoietic cells 130.

These studies emphasize the crucial roles of 8 GPCRs in processes such as tumor cell behavior, insulin resistance, immune response, fracture healing, and bone metastasis (Table 5). These findings enhance our understanding of various biological processes and diseases, suggesting GPCRs as potential therapeutic targets and prognostic markers.

## Therapeutic applications of GPCR-targeting drugs in bone disorders

**Figure 6:**
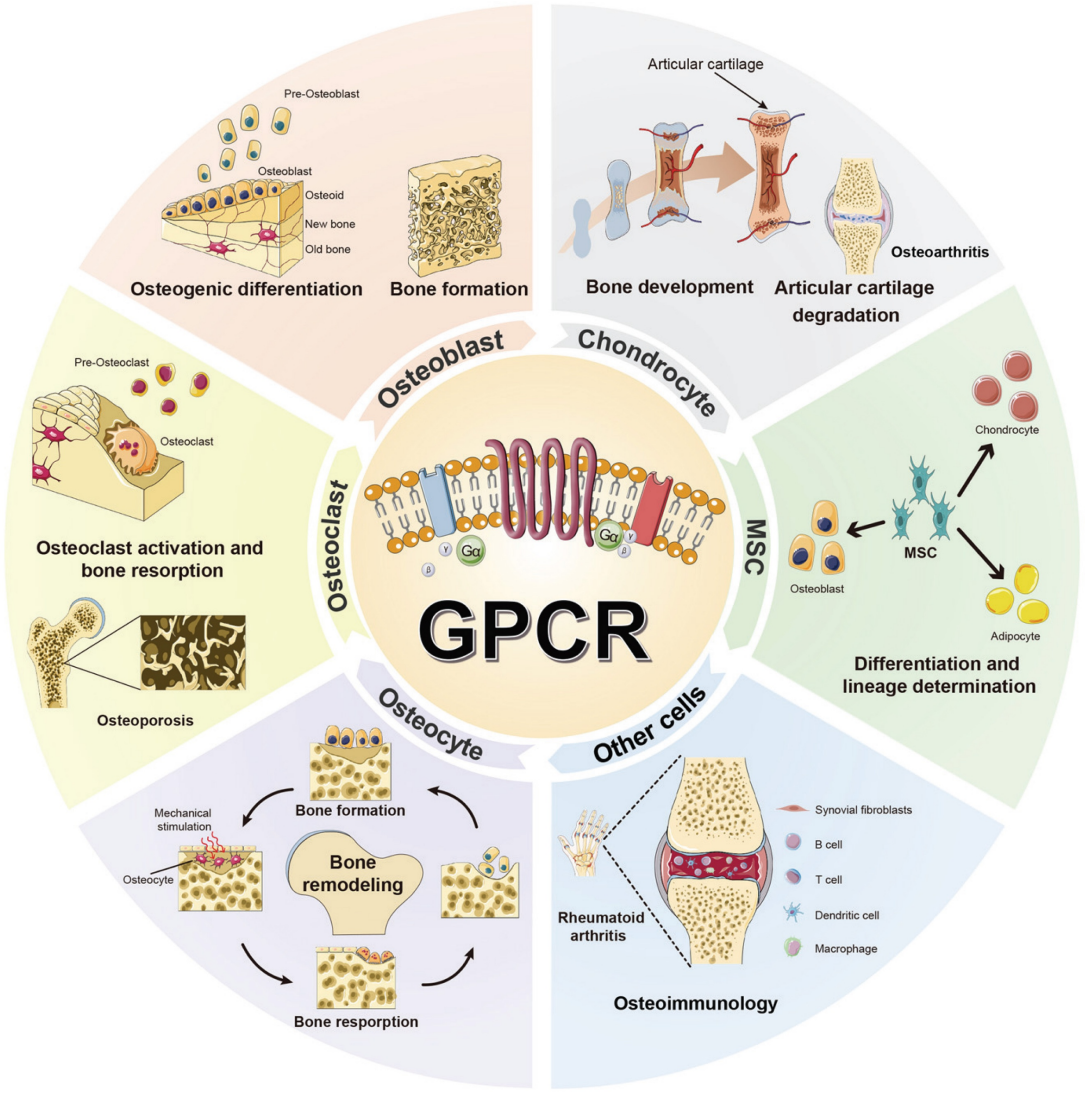
The function of GPCRs in different bone cells.

The development of GPCR-targeted therapeutics has maintained a pioneering role in biomedical innovation. Currently, therapeutic agents engaging these receptors represent a critical component of modern clinical practice, with ongoing expansion observed in both drug discovery pipelines and experimental therapeutic programs (Table 6). This progression reflects the established importance of GPCR modulation across multiple therapeutic domains. The evolving landscape of GPCR-based interventions underscores their enduring relevance as drug development targets. Building on mechanistic insights into GPCR-orchestrated cellular processes in bone biology—including osteoblast differentiation, osteoclast activity modulation, and paracrine signaling during remodeling—the therapeutic rationale for targeting these receptors has gained substantial traction. Dysregulation of GPCR signaling pathways, manifesting through aberrant ligand interactions, receptor desensitization, or imbalanced downstream effector cascades, forms the pathophysiological basis for skeletal disorders such as osteoporosis, rheumatoid arthritis, and osteogenesis imperfecta. GPCRs represent a critical class of therapeutic targets in bone disorders, offering significant clinical value and research potential due to their widespread tissue distribution and diverse signal transduction mechanisms (Fig. 6). These receptors enable precise modulation of bone metabolism through multiple intervention points, forming a continuum of drug development from approved medications to innovative investigational agents.

Among FDA-approved therapies, PTH1R agonists have established a paradigm shift in anabolic bone treatment 4, 7. Teriparatide (PTH1-34) exemplifies this class through non-selective activation of Gαs/cAMP and Gαq pathways, enhancing osteoblast activity while necessitating careful monitoring for osteosarcoma risks. This challenge has driven the development of next-generation agents like abaloparatide, a biased agonist that preferentially activates Gαs-mediated osteoanabolic signals, maintaining efficacy with improved safety profiles 55. Both medications hold FDA approval for managing severe osteoporosis in high-fracture-risk populations.

CaSR modulators constitute another therapeutic pillar. Calcimimetics such as cinacalcet and evocalcet enhance CaSR sensitivity to extracellular calcium, effectively suppressing PTH hypersecretion and stabilizing bone turnover in secondary hyperparathyroidism and chronic kidney disease-related bone disorders 6, 24. Notably, evocalcet demonstrates reduced gastrointestinal adverse effects through enhanced receptor selectivity. Emerging as a complementary approach, calcilytics (CaSR antagonists) are being investigated as intermittent endogenous PTH stimulators with unique bone-forming potential in preclinical models 6, 15.

Exploration of novel targets has revealed multidimensional regulatory roles for GPCRs in bone metabolism. CB2 agonists exhibit dual regulatory capacity, inhibiting osteoclastogenesis while promoting osteogenesis in osteoporosis models and protecting against cancer-induced bone destruction in metastatic settings 81, 117. Adenosine receptor modulation demonstrates therapeutic synergy, with A2A agonists simultaneously enhancing osteoblast differentiation and suppressing osteoclast formation, while A2B signaling directly augments bone matrix mineralization. Pulsed electromagnetic fields further amplify this system's therapeutic potential by upregulating A2A/A3 receptor expression, creating synergistic bone regeneration effects 53.

Precision signaling modulation has become the vanguard of next-generation drug design. Biased PTH1R agonists exemplify this strategy by selectively activating anabolic pathways while minimizing unwanted bone resorption signals 7. Calcium-sensing receptor allosteric modulators are engineered for skeletal-specific responses, and β-adrenergic receptor regulators are being optimized to modulate osteoblast-osteoclast communication with maximal target specificity 4, 5. These approaches reflect a paradigm shift from traditional orthosteric agonists to more nuanced signal control mechanisms.

Therapeutic boundaries continue expanding across disease spectra. CXCR4 antagonism, already validated for stem cell mobilization, demonstrates preclinical efficacy in blocking tumor cell bone homing through CXCL12-CXCR4 axis disruption 129. GPR40/GPR120 agonists exhibit chondroprotective and anti-inflammatory effects in osteoarthritis models 102, 106, while GPR41/GPR43 activation mediates immunometabolic regulation with dual benefits in metabolic bone diseases 131. Notably, GLP-1R/GCGR dual agonists achieve synergistic osteoanabolic and metabolic control in diabetes-associated osteoporosis 132.

Technological advancements are accelerating therapeutic innovation. Cryo-electron microscopy structures of B-class GPCR-G protein complexes provide atomic-level templates for biased ligand design. Single-cell transcriptomics uncovers skeletal cell heterogeneity, enabling targeted drug delivery systems. AI-driven platforms expedite the discovery of multifunctional molecules, though challenges persist in managing signaling cross-talk, ensuring long-term safety, and optimizing precision strategies for age-specific populations.

As the field progresses, GPCR modulation is transitioning from single-target interventions to network-based precision therapies. This evolution holds transformative potential across osteoporosis, osteoarthritis, skeletal metastases, and inflammatory bone diseases, heralding a new era of skeletal medicine grounded in molecular precision and therapeutic innovation.

## Emerging concepts in GPCR research

The evolution of GPCR research has profoundly impacted bone biology, offering transformative insights into skeletal physiology and therapeutic strategies. Recent advancements in structural biology, signaling modulation, and spatial omics are reshaping our understanding of GPCRs as key regulators of bone metabolism, with direct implications for diseases like osteoporosis and osteoarthritis. This review highlights five pivotal concepts bridging GPCR innovation to bone health, emphasizing translational relevance.

### Structural revelations via Cryo-EM: unlocking class B/C GPCR mechanisms

Cryo-EM has revolutionized structural elucidation of bone-relevant GPCRs. For class B receptors, the high-resolution structure of PTH1R - a master regulator of calcium homeostasis - revealed how PTH stabilizes transmembrane helix 6 to activate Gs proteins, providing a molecular template for osteoporosis drug design 133, 134. Similarly, class C GABAB receptor heterodimer structures demonstrated asymmetric activation: GB1 subunit binds ligands while GB2 couples to Gi proteins, offering mechanistic insights into pathways disrupted in skeletal dysplasias 135-137. These structures not only clarify ligand-receptor interactions but also enable rational design of biased agonists and allosteric modulators that selectively target bone-specific GPCR conformations.

### Biased signaling and allosteric modulation: precision control of bone remodeling

Allosteric modulators, binding outside orthosteric sites, introduce therapeutic advantages by fine-tuning receptor activity without desensitization 138, 139. In bone, PCO371 exemplifies this paradigm: by stabilizing PTH1R's intracellular transducer pocket, it activates Gs signaling independent of extracellular ligand binding, bypassing traditional desensitization pathways 139-141. Such biased allosteric modulators direct signaling toward G protein-mediated anabolic pathways (e.g., osteoblast stimulation) while avoiding β-arrestin-driven catabolic effects (e.g., osteoclast activation) 142-145. This precision is critical for osteoporosis, where maintaining balanced bone turnover remains challenging.

### GPCR heterodimerization: emerging regulatory paradigms in bone cells

Heterodimerization expands GPCR functional diversity beyond monomeric paradigms 146-148. The GABAB receptor heterodimer exemplifies this principle, where subunit cooperation enables nuanced signaling control. In bone, heterodimers like CXCR4-CXCR7 may integrate chemokine gradients to regulate osteoclast migration, while CaSR dimers modulate osteoblast differentiation. Targeting these complexes offers dual therapeutic angles: enhancing protective heterodimers or disrupting pathogenic ones. For instance, stabilizing osteoprotective mGluR2-mGlu4 heterodimers could mitigate aberrant resorption in rheumatoid arthritis 143, 149.

### Endosomal signaling: subcellular control of bone homeostasis

Traditional views of plasma membrane-restricted GPCR signaling are evolving with discoveries of endosomal persistence 150-152. The PTH1R-β-arrestin1 complex, for example, sustains Gs signaling from endosomes to regulate calcium fluxes long after surface receptor internalization 153-155. This spatial redistribution explains PTH's dual anabolic (acute) and catabolic (chronic) effects in bone, where prolonged endosomal signaling may drive pathological remodeling. Therapeutically, manipulating endosomal trafficking - via dynamin inhibitors or lysosomal escape modulators - could optimize PTH analogs for sustained anabolic benefits in osteoporosis 156-160.

### Single-cell GPCR atlas: mapping heterogeneity in skeletal niches

Single-cell RNA sequencing (scRNA-seq) is resolving GPCR expression heterogeneity across bone cell lineages. Preliminary studies in immune cells highlight how neuroinflammatory conditions alter GPCR profiles in osteoclast precursors, but bone-centric analyses remain sparse 161. Future efforts should profile osteoblasts, osteocytes, and osteoclasts under pathological states (e.g., aging, diabetes) to identify disease-specific GPCR signatures. Such atlases would enable targeted delivery of GPCR modulators - such as CX3CR1 agonists to polarize osteoclasts toward anti-inflammatory phenotypes - while sparing off-target tissues.

Modern GPCR research converges on bone biology through structural precision, signaling refinement, and spatial omics. Cryo-EM-guided drug design, biased allosteric modulation, heterodimer targeting, endosomal signaling manipulation, and single-cell GPCR mapping collectively address unmet needs in skeletal medicine. As these technologies mature, the vision of precision-engineered GPCR therapeutics - from PTH1R-biased agonists to osteocyte-specific allosteric modulators - moves closer to clinical reality, offering new hope for fractured bones and fragile lives.

## Perspective and Conclusion

GPCRs represent highly promising targets for pharmacological intervention due to their widespread distribution and involvement in numerous physiological processes 9, 121. This review systematically consolidates the roles of GPCRs across bone cell types: 12 GPCRs in MSCs, 21 in osteoblasts and osteocytes, 23 in macrophages and osteoclasts, 31 in chondrocytes, and 8 in other bone-related cells (Tables 1-5).

These GPCR receptors are functionally categorized by their coupled Gα subunits (Fig. 3). Gαs-coupled receptors (e.g., PTH1R, A2AR, MC1R/MC3R) promote osteogenesis or suppress osteoclast activity via cAMP/PKA signaling, as exemplified by PTH1R-driven RUNX2 activation in osteoblasts, a mechanism harnessed clinically by the osteoporosis drug teriparatide. Conversely, ADRB2 in chondrocytes paradoxically inhibits differentiation markers through ERK1/2-PKA, underscoring cell-type specificity. Gαi/o-coupled receptors (e.g., GPR120, A1R, CNR2) primarily inhibit osteoclastogenesis or inflammation, with GPR41 enhancing insulin sensitivity in diabetic models via calcium signaling. Gαq-coupled receptors (e.g., CaSR, PAR2, EDG2) regulate bone metabolism and pathologies such as tumor metastasis through PLC/PKC or NF-κB pathways—renal cancer cells overexpressing CaSR, for instance, exhibit heightened bone metastatic potential. Gα12/13-coupled GPCRs like GPR55 drive osteoclastogenesis via Rho/ROCK, highlighting their role in bone resorption (Table 1-5).

GPCR activity is influenced by age, genetic, and environmental factors, with functional changes directly contributing to bone metabolic imbalances during development, aging, and disease progression. The functional diversity of GPCRs arises from pathway and cell-type specificity (Table 1-5). For example, ADRB2 enhances RANKL expression in osteoblasts to amplify osteoclast activity, yet suppresses chondrocyte differentiation markers, illustrating context-dependent roles. In osteoblasts, core pathways include Gαs/cAMP-PKA and Gαq/PLC-PKC: PTH1R activates RUNX2 and Osterix to stimulate bone formation, while CaSR boosts mineralization via AKT-β-catenin (Fig. 4 and Fig. 5). Osteoclasts rely on Gαq/PLC-IP3-Ca²⁺ and Gαi/EBI2-RhoA pathways, where CaSR activates NFATc1 to upregulate bone-resorbing enzymes (TRAP, CTSK), and EBI2 directs precursor migration to bone surfaces. MSCs balance osteogenic and adipogenic differentiation through Gαs/Wnt-β-catenin and Gβγ/MAPK signaling—LGR5 promotes osteogenesis, whereas ADRB2 suppresses differentiation via cAMP. Chondrocyte homeostasis hinges on Gαi/CXCR4-ERK and Gαq/GPR120-NF-κB pathways: CXCR4 drives hypertrophic differentiation, while GPR120 preserves cartilage matrix by inhibiting IL-1β-induced collagen degradation.

Therapeutically, GPCRs offer versatile targets. Gαs agonists (e.g., A2AR agonists, PTH1R activators) enhance bone regeneration, while Gαi agonists like GPR120 ligands suppress osteoclast activity in inflammatory bone loss. Gαq antagonists such as the PAR2 inhibitor AZ3451 mitigate osteoarthritis progression by blocking cartilage degradation. In cancer, targeting GPRC5A or CaSR may inhibit bone metastasis, whereas GPR41 activation improves glucose metabolism in metabolic disorders. Beyond single-cell functions, GPCRs integrate mechanical, metabolic, and immune signals: mechanical sensors like Gpr161 and Piezo1 guide MSC differentiation under stress; metabolic GPCRs (e.g., GPR41) link energy status to bone remodeling; and immune-bone crosstalk involves EP2/EP4-PGE2 (anti-inflammatory) and H4R (pro-osteoclastogenic in rheumatoid arthritis). For instance, CXCR4/SDF-1 and PAR2/NF-κB pathways exacerbate osteoarthritis through cartilage hypertrophy and matrix degradation, while MC1R/MC3R and GPR120/SOX9 counteract inflammation to preserve cartilage.

Despite their therapeutic promise, critical gaps persist. Most studies focus on GPCR roles in isolated cell types, neglecting systemic coordination across osteoblasts, osteoclasts, and immune cells (Fig. 6). For example, bone formation-resorption equilibrium likely requires GPCR-mediated dialogue between these lineages, yet such networks remain poorly mapped. Additionally, disease-stage-specific GPCR dynamics—such as CaSR's dual roles in early inflammation versus late fibrosis in osteoarthritis—are underexplored, obscuring precise therapeutic windows. Overcoming these challenges demands longitudinal studies of GPCR crosstalk and temporal regulation, leveraging advances in single-cell omics and biased ligand design. By bridging these gaps, GPCR-targeted therapies could revolutionize treatment for osteoporosis, osteoarthritis, and metastatic bone diseases, transforming mechanistic insights into clinical breakthroughs.

Moreover, the journey from bench to bedside of GPCR targeted therapies is fraught with complexity. The translation of GPCRs research into effective clinical treatments for bone diseases presents several challenges 4, 5.

### Complexity of GPCR signaling

GPCRs can activate multiple signaling pathways, which can have diverse and sometimes opposing effects on bone metabolism. This signaling complexity can cause unpredictable outcomes when GPCRs are targeted therapeutically. For example, while one pathway may promote bone formation, another might simultaneously enhance bone resorption. Dissecting these pathways for selective targeting is a major challenge. To address the complexity of GPCR signaling in therapeutic development, integrated strategies should combine pathway-selective modulation with emerging technologies. First, biased agonists that preferentially activate G protein- versus β-arrestin-dependent pathways could decouple therapeutic effects from adverse liabilities, as demonstrated by μ-opioid receptor ligands separating analgesia from respiratory depression 3. Complementary approaches include allosteric modulators and nanobody-based tools to stabilize functional receptor conformations or disrupt pathological oligomerization complexes, enabling spatial control over signaling microenvironments. Temporal regulation may be achieved through targeted protein degradation systems like PROTACs, which dynamically adjust receptor surface density to mitigate desensitization in chronic disorders, or CRISPR-based epigenome editing to permanently correct transcriptional imbalances in genetic conditions 162, 163. Multi-omics integration offers another dimension, with single-cell analytics and machine learning identifying patient-specific signaling signatures to guide precision ligand combinations. For localized interventions, optogenetic GPCR variants provide non-invasive 164, spatiotemporally precise control over bone remodeling processes, enabling circadian rhythm restoration in osteoblast-osteoclast coupling or acute suppression of inflammatory cascades in arthritic joints.

### Pleiotropic effects

Many GPCRs play roles in multiple physiological processes beyond bone metabolism 1, 7. Targeting these receptors can therefore lead to unintended side effects in other tissues or systems. For instance, the cannabinoid receptors CB1 and CB2, which influence bone metabolism, are also involved in neurological and immune system functions 125. This pleiotropy complicates the development of therapies that are both effective and safe.

### Drug specificity and selectivity

Developing drugs that specifically target bone-related GPCRs without affecting similar receptors in other tissues is challenging. The high degree of homology among GPCR family members complicates the creation of highly selective ligands 4, 5. Non-specific activation or inhibition of GPCRs can lead to off-target effects, reducing the therapeutic utility of a potential drug.

### Limitations in models

*In vitro* experiments are usually carried out in cell lines or purified receptor systems, lacking the complex physiological environment *in vivo*, including interactions between cells, tissue specificity, and various regulatory factors in the body. In addition, in the heterologous expression system, the expression level of GPCRs may differ from that under physiological conditions *in vivo*. This may lead to changes in the aggregation state of the receptors, ligand-binding characteristics, and signal transduction functions.

When studying the structure and function of GPCRs *in vivo*, due to the lack of effective real-time monitoring techniques, it is difficult to directly observe the dynamic changes of the receptors under physiological conditions. Moreover, the genetic background of animal models may affect the observation and analysis of GPCR-related phenotypes. Some unexpected genetic changes may be introduced during the gene editing process, thus interfering with the accurate evaluation of the functions of GPCRs.

### Pharmacological desensitization and tachyphylaxis

Repeated stimulation of GPCRs can lead to desensitization, where the receptor becomes less responsive to its ligand. This phenomenon can undermine the long-term effectiveness of GPCR-targeted therapies, as seen with certain therapies for osteoporosis that use PTH analogs like teriparatide, abaloparatide and romosozumab 7. Managing receptor desensitization and developing strategies to overcome it are significant hurdles.

Despite these challenges, the therapeutic potential of targeting GPCRs in bone diseases remains significant. Advances in molecular biology, Structural biology, pharmacology, and drug design are gradually overcoming these hurdles. The development of Cryo-EM has provided us with a clearer understanding of the molecular structure of GPCRs, laying the foundation for the study of their more complex molecular functions 165, 166. Based on this, research on biased agonists may bring breakthroughs in the treatment of precisely targeting GPCR signaling pathways. In addition, emerging concepts such as GPCR heterodimers and GPCR spatio-temporal signaling may lead to the discovery of more GPCR drug targets. Research on GPCR expression in single - cell transcriptomes of the skeletal niches may provide new perspectives for the study of the synergistic effects of GPCR signaling among different bone cells. Continued research, interdisciplinary collaboration, and innovative approaches are essential to harness the full therapeutic potential of GPCRs in treating bone diseases. Future research should focus on elucidating the cross-talk between different GPCR signaling pathways and their integration with other signaling cascades to fully harness the potential of GPCRs in modulating bone cells behavior and bone health.

## Figures and Tables

**Table 1:** The functions and mechanisms of GPCR in mesenchymal stem cells (MSCs)

GPCR Name	GRAFS Classification	Ligand	Coupled G Protein Subtype	Signaling Pathway	Functional/Phenotypic Changes and references
A1R (ADORA1)	Rhodopsin family - α subgroup (Amines)	Adenosine	Gαi	WNT signaling	Stimulates osteogenic differentiation in dental pulp stem cells 17.
A2AR (ADORA2A)	Rhodopsin family - α subgroup (Adenosine)	Adenosine	Gαs	cAMP/PKA	Increases proliferation and differentiation of bone marrow MSCs 18.
ADORA2B (A2BAR)	Rhodopsin family - α subgroup (Adenosine)	Adenosine	Gαs	Osteoblast differentiation gene regulation	Deletion lowers bone mineral density and reduces mineralization in BMSCs 19.
ADRB2 (β-AR)	Rhodopsin family - α subgroup (Adrenergic)	Norepinephrine, Isoproterenol, Epinephrine	Gαs	cAMP/PKA	Agonists inhibit mineralization and osteogenesis; antagonists promote mineralization 22, 23.
CaSR	Glutamate family	Extracellular Ca²⁺	Gαq/Gαi	Calcium sensing → PTHrP/PTH1R inhibition	Maintains osteogenic potential; knockdown reduces bone formation capacity 15.
EP1	Rhodopsin family	PGE2	Gαq/11	↑ PLCβ → IP₃/DAG → Ca²⁺ release, PKC activation	Inhibits osteoblast migration, exacerbates osteoarthritis 20, 21.
EP2/EP4 (PTGER)	Rhodopsin family	PGE2	Gαs	FAK/ERK1/2 → PGE2 release	Enhances MSC migration; boosts immunosuppressive effects on RA T cells 27, 28.
GPR161	Other 7TM receptors	/	Gαs	Mechanical force → cAMP signaling	Knockdown impairs mechanotransduction and reduces osteogenic marker expression 29.
GPRC6A	Glutamate family	cation, amino acid, and testosterone	Gαq (putative)	Extracellular calcium → ERK activation	Promotes osteogenic marker expression and mineralization; knockout reduces bone mineral density 16.
LGR5 (GPR49)	Adhesion family	R-spondin	Gαs (putative)	Wnt/β-catenin, ERK, mitochondrial dynamics	Promotes osteogenic differentiation of MSCs; overexpression accelerates fracture healing 14.
LPAR1 (EDG2/GPCR26)	Rhodopsin family - δ subgroup (Lipids)	Lysophosphatidic acid, LPA	Gα12/Gα13 (major), Gαq/Gαi (minor)	Caspase-3 inhibition → anti-apoptosis	Protects MSCs from apoptosis; promotes proliferation 26.
PTH1R	Secretin family	PTH/ PTHrP	Gαs	cAMP/PKA → Runx2/osteocalcin	Enhances osteogenic differentiation and bone formation; inhibits osteoclastogenesis 6, 15.

**Table 2:** The functions and mechanisms of GPCR in osteoblasts and osteocytes

GPCR Name	GRAFS Classification	Ligand	Coupled G Protein Subtype	Signaling Pathway	Functional/Phenotypic Changes and references
A1R (ADORA1)	Rhodopsin family - α subgroup (Amines)	Adenosine	Gαi	Adipogenic signaling	Promotes adipogenesis over osteoblast differentiation 50.
A2AR (ADORA2A)	Rhodopsin family - α subgroup (Adenosine)	Adenosine	Gαs	cAMP/PKA	Stimulates osteoblast activity and suppresses osteoclast activity; promotes bone regeneration 50, 52.
A3AR (ADORA3)	Rhodopsin family - α subgroup (Adenosine)	Adenosine	Gαi	Anti-inflammatory signaling	Reduces inflammatory cytokines (e.g., TNF-α); promotes bone repair in inflammatory conditions 53.
ADORA2B	Rhodopsin family - α subgroup (Adenosine)	Adenosine	Gαs	cAMP/PKA	Inhibits adipogenesis and stimulates osteoblast differentiation 51.
ADRA1A (α1-AR)	Rhodopsin family - α subgroup (Adrenergic)	Epinephrine, Norepinephrine, Phentolamine	Gαq	/	Negatively regulates Bmp4 expression by up-regulating Nfil3/E4BP4 in osteoblasts 41.
ADRA2A (α2A-AR)	Rhodopsin family - α subgroup (Adrenergic)	Epinephrine, Norepinephrine, Phentolamine	Gαi	Neuro-endocrine signaling	SNP rs553668 and rs1800544 locate in gene; alters mRNA stability and BMD 42.
ADRB1	Rhodopsin family - α subgroup (Adrenergic)	Norepinephrine, isoproterenol	Gαs	cAMP/PKA	Inhibits disuse-induced bone loss by reducing osteocyte apoptosis 37.
ADRB2	Rhodopsin family - α subgroup (Adrenergic)	Norepinephrine, Isoproterenol, Epinephrine	Gαs	cAMP/PKA → RANKL upregulation	Deficiency increases bone mass 38-40.
CaSR	Glutamate family	Extracellular Ca²⁺	Gαq/Gαi	mTORC2/AKT-β-catenin, NF-κB/JAK-Stat3	Promotes bone formation (via β-catenin); drives pathological bone formation in inflammatory states 34.
CNR2 (CB2)	Rhodopsin family - α subgroup (Cannabinoid)	Endocannabinoids, CB2 agonists	Gαi	Osteoclast survival suppression	Agonists promote osteoblast differentiation and protect against ovariectomy-induced bone loss 55, 56.
EP2	Rhodopsin family	PGE2	Gαs	cAMP/PKA	Enhances osteoblast differentiation and mineralization 49.
GABABR	Glutamate family	GABA	Gαi	cAMP suppression → BMP2/RANKL downregulation	Inhibits osteoblastogenesis; knockout increases BMP2 expression but reduces BMD 45.
GPR161	Other 7TM receptors	/	Gαi (putative)	Sonic Hedgehog (Shh) pathway suppression	Inhibits intramembranous bone formation; knockout completely blocks osteoblastogenesis 29, 46.
GPR39	Other 7TM receptors	Zinc	Gαq	Unknown (matrix deposition regulation)	Knockout causes disordered matrix deposition (low collagen, high mineral content) 36.
GPR48 (LGR4)	Adhesion family	R-spondin	Gαs	cAMP-PKA-Atf4	Regulates embryonic osteoblast differentiation and mineralization; knockout delays bone formation 31.
GPRC6A	Glutamate family	Calcium/Amino acids	Gαq	ERK activation	Suppresses osteoblast differentiation and ALP activity; knockout reduces bone formation 16.
GRM1 (mGluR1)	Glutamate family	Glutamate	Gαq	/	Knockout causes premature growth plate fusion and osteoblast dysfunction 48.
LGR5 (GPR49)	Adhesion family	R-spondin	Gαs	Wnt/β-catenin activation	Promotes osteoblast differentiation and bone formation 30.
LPAR1 (EDG2/GPCR26)	Rhodopsin family - δ subgroup (Lipids)	Lysophosphatidic acid (LPA)	Gα12/13	RANKL→MAPK/AKT-NF-κB	Knockout reduces bone mineralization, increases osteocyte apoptosis, and bone porosity 47.
PTH1R	Secretin family	PTH/ PTHrP	Gαs	cAMP/PKA	Activation (e.g., by teriparatide) stimulates bone formation; key therapeutic target for osteoporosis 6, 7.
XCR1	Other 7TM receptors	XCL1	Gαi (putative)	/	Promotes osteoblast differentiation and enhances bone formation 54.

**Table 3:** The functions and mechanisms of GPCR in osteoclasts

GPCR Name	GRAFS Classification	Ligand	Coupled G Protein Subtype	Signaling Pathway	Functional/Phenotypic Changes and references
A1R (ADORA1)	Rhodopsin family - α subgroup (Amines)	Adenosine	Gαi	Pro-osteoclast signaling	Promotes osteoclast formation and bone loss 74.
A2AR (ADORA2A)	Rhodopsin family - α subgroup (Adenosine)	Adenosine	Gαs	cAMP/PKA	Inhibits osteoclast differentiation; agonists promote bone regeneration 74.
ADRB2	Rhodopsin family - α subgroup (Adrenergic)	Norepinephrine, Isoproterenol, Epinephrine	Gαs	RANKL upregulation	Enhances osteoclastogenesis and bone resorption 38-40.
CaSR	Glutamate family	Extracellular Ca²⁺	Gαq/Gαi	NF-κB, Akt	High Ca²⁺ inhibits osteoclast resorption; osteoblast knockout increases RANKL-driven resorption 67-71.
CNR1 (CB1)	Rhodopsin family - α subgroup (Cannabinoid)	Endocannabinoids, e.g., anandamide	Gαi	Apoptosis induction	Antagonists increase bone mass by promoting osteoclast apoptosis 78.
CNR2 (CB2)	Rhodopsin family - α subgroup (Cannabinoid)	Endocannabinoids, CB2 agonists	Gαi	Osteoclast survival suppression	Agonists promote osteoclast formation; antagonists inhibit bone loss 81.
DRD2 (D2DR)	Rhodopsin family - α subgroup (Dopamine)	Dopamine	Gαi	NF-κB suppression	Inhibits M1 macrophage polarization; restricts inflammatory osteolysis 82.
EBI2 (GPR183)	Rhodopsin family - γ subgroup (Chemokine)	7α,25-OHC	Gαi	7α,25-OHC → OCP migration/fusion	Promotes osteoclast precursor migration; defective signaling increases bone mass 58.
EP4 (PTGER4)	Rhodopsin family	PGE2	Gαs	PGE2 → cytokine-driven resorption	Enhances osteoclast formation in inflammation; overexpression inhibits resorption 85-87.
F2r (Thrombin Receptor)	Rhodopsin family - δ subgroup (Thrombin)	Thrombin	Gαq/11	Inhibiting Akt-GSK3β-NFATc1 and suppressing NF-κB signaling	Inhibits osteoclast formation and bone resorption 60.
GABABR	Glutamate family	GABA	Gαi	cAMP suppression → BMP2/RANKL downregulation	Knockout elevates ALP levels and BMP2/Osterix expression, which inhibits osteoclast formation by reducing RANKL production 45.
GPR120 (FFAR4)	Rhodopsin family - α subgroup (Fatty Acid)	Long-chain fatty acids	Gαq/Gαq11	ROS suppression → antioxidant activation	Inhibits osteoclast formation/resorption; reduces ROS production 65.
GPR125	Adhesion family	/	Gαq/12/13 (putative)	RANKL → MAPK/AKT-NF-κB	Promotes osteoclast differentiation/activation; knockdown reduces signaling 84.
GPR132 (G2A)	Other 7TM receptors	Lysophosphatidic acid, LPA	Gα12/13	Macrophage polarization	Reduces M1-like macrophage infiltration; shifts to M2 polarization 64.
GPR30	Other 7TM receptors	Estrogen	Gαi (putative)	Membrane estrogen signaling	Inhibits osteoclastogenesis via non-nuclear pathways 61.
GPR48 (LGR4)	Adhesion family	R-spondin	Gαs (putative)	cAMP-PKA-CREB → Atf4	Delays embryonic osteoblast differentiation; postnatal knockout increases osteoclast activity 62, 63.
GPR54	Other 7TM receptors	Kisspeptin	Gαq (putative)	Kp-10 → Dusp18/Src dephosphorylation	Suppresses osteoclast activity; prevents bone loss 66.
GPR55	Other 7TM receptors	Lysophosphatidic acid, LPA	Gα12/13 (putative)	RANKL → NFATc1 activation	Enhances osteoclast maturation; inhibition reduces bone resorption 59.
GPRC6A	Glutamate family	Calcium/Amino acids	Gαq (putative)	ucOCN-mediated inhibition	Inhibits early osteoclast differentiation and resorption 72.
H4R (GPCR105)	Rhodopsin family - α subgroup (Histamine)	Histamine	Gαi (putative)	RANKL upregulation	Promotes RA-associated osteoclastogenesis; antagonists reduce bone destruction 88.
LPAR1 (EDG2/GPCR26)	Rhodopsin family - δ subgroup (Lipids)	Lysophosphatidic acid, LPA	Gα12/13	RANKL → MAPK/AKT-NF-κB	Essential for osteoclast differentiation; antagonists inhibit resorption 83.
TAS1R3	Glutamate family	Sweet tastants	Gαi (putative)	Nutrient sensing	Increases cortical bone mass; reduces osteoclast activity 73.
TSHR (LGR3)	Rhodopsin family - δ subgroup (Glycoprotein)	TSH	Gαs	TSH → cAMP/PKA	Inhibits osteoclastogenesis; knockout reduces bone strength 57.

**Table 4:** The functions and mechanisms of GPCR in chondrocytes

GPCR Name	GRAFS Classification	Ligand	Coupled G Protein Subtype	Signaling Pathway	Functional/Phenotypic Changes and references
A2AR	Rhodopsin family - α subgroup (Adenosine)	Adenosine	Gαs	FoxO/autophagy activation	Stimulation improves cartilage function; enhances autophagy and reduces inflammation 114.
A3AR (ADORA3)	Rhodopsin family - α subgroup (Adenosine)	Adenosine	Gαi	Suppression of RUNX2/CaMKII	Agonists inhibit matrix degradation and cartilage hypertrophy in OA 108.
ADORA2B	Rhodopsin family - α subgroup (Adenosine)	Adenosine	Gαs	cAMP/PKA	Activation inhibits chondrogenic differentiation in MSCs by downregulating SOX9 and COL2A1 50.
ADRA1A (α1-AR)	Rhodopsin family - α subgroup (Adrenergic)	Epinephrine, Norepinephrine, Phentolamine	Gαq	ERK/PKA	Low-dose NE induces apoptosis via α1-AR; accelerates OA pathogenesis 113.
ADRA2A (α2A-AR)	Rhodopsin family - α subgroup (Adrenergic)	Epinephrine, Norepinephrine, Phentolamine	Gαi	ERK1/2-PKA/cGMP	Activation ↑ MMPs and RANKL, causing cartilage degeneration; antagonists as enhancers of chondrogenesis and suppressors of hypertrophy, agonists induced detrimental hypertrophy 111.
ADRB2 (β-AR)	Rhodopsin family - α subgroup (Adrenergic)	Norepinephrine, Isoproterenol, Epinephrine	Gαs	ERK1/2-PKA/Jun-B	Agonists inhibit chondrocyte differentiation markers; high-dose NE reverses IL-1β damage 109, 110.
AT1R	Rhodopsin family	Angiotensin II	Gαq	ERK/PKA	High AT1R/AT2R ratio impedes chondrocyte proliferation under stress; inhibition promotes survival 115.
AT2R	Rhodopsin family	Angiotensin II	Gαi (putative)	Counteracts AT1R	Enhanced expression reduces apoptosis in stressed chondrocytes 115.
CaSR	Glutamate family	Extracellular Ca²⁺	Gαq	Pro-differentiation signaling	Biomechanical stress ↑ CaSR expression, accelerating OA; calcilytics block cartilage degradation 15, 24, 33.
CNR1 (CB1)	Rhodopsin family - α subgroup (Cannabinoid)	Endocannabinoids, e.g., anandamide	Gαi	SIRT1 activation	Agonists protect against IL-1β-induced senescence and cell cycle arrest 117.
CNR2 (CB2)	Rhodopsin family - α subgroup (Cannabinoid)	Endocannabinoids, CB2 agonists	Gαi	Anti-inflammatory	Deficiency worsens OA; agonists reduce OA severity and enhance proteoglycan synthesis 117.
CCR2	Rhodopsin family - γ subgroup (Chemokine)	CCL2	Gαi	NF-κB/MAPK	Drives macrophage recruitment and cartilage erosion in OA; antagonism reduces synovitis and damage 93.
CCR5	Rhodopsin family - γ subgroup (Chemokine)	CCL3/CCL4/CCL5	Gαi	/	Deficiency protects against cartilage degeneration in OA 92.
CXCR2	Rhodopsin family	CXCL1/CXCL8	Gαi	AKT signaling	Maintains chondrocyte homeostasis; knockout increases osteoarthritis severity (↑ apoptosis, ↓ ECM) 90.
CXCR3	Rhodopsin family	CXCL9/CXCL10/CXCL11	Gαi	ER stress (CHOP/GRP78)	Elevated in OA; siRNA knockdown reduces nitrate-induced chondrocyte apoptosis 91.
CXCR4	Rhodopsin family	CXCL12	Gαi	SDF-1/CXCR4-Runx2 feedback loop	Promotes chondrocyte hypertrophy; blocking CXCR4 inhibits hypertrophy and delays growth plate closure 89.
EP1	Rhodopsin family	PGE2	Gαq	PGE2 signaling	Inhibits fracture healing; EP1 knockout accelerates bone repair 21.
EP2	Rhodopsin family	PGE2	Gαs	cAMP/PKA	Suppresses MMP-13 (anti-catabolic); combined EP2/EP4 activation mimics PGE2-induced collagen synthesis 100.
EP4	Rhodopsin family	PGE2	Gαs	cAMP/PKA	Cooperates with EP2 to regulate chondrocyte differentiation and matrix synthesis 21, 99.
GLP-1R	Secretin family	GLP-1	Gαs	PI3K/Akt/NF-κB	Activation reduces ER stress, apoptosis, and inflammation; attenuates OA cartilage degeneration 132, 167.
GPR120	Rhodopsin family - α subgroup (Fatty Acid)	Long-chain fatty acids	Gαq	SOX9-mediated ECM protection	Agonists rescue type II collagen and aggrecan expression; suppresses IL-1β-induced ECM loss 106.
GPBAR1	Rhodopsin family	Bile acids	Gαs	Anti-senescence	Protects chondrocytes from IL-1β-induced senescence; activation reduces β-galactosidase activity 104.
GPR4	Rhodopsin family	Protons	Gα12/13 or Gαq	NF-κB/MAPK	Drives OA progression; knockout or inhibition attenuates cartilage degradation 105.
GPR40	Rhodopsin family	Medium/long-chain fatty acids	Gαq (putative)	NF-κB inhibition	Agonists reduce matrix-degrading enzymes and inflammation; slows OA progression 102.
GPR43	Rhodopsin family	Short-chain fatty acids, e.g., propionate	Gαi	Anti-inflammatory signaling	Activated by butyrate; mitigates IL-1β-induced MMPs and collagen degradation 103.
GPR84	Rhodopsin family	Medium-chain fatty acids	Gαi	NF-κB inhibition	Deficiency ↑ cartilage catabolism; activation blocks IL-1β-induced OA pathogenesis 107.
H4R (GPCR105)	Rhodopsin family - α subgroup (Histamine)	Histamine	Gαi	cAMP↓, MAPK↑	Linked to hypertrophic chondrocyte differentiation (co-expressed with COLX) 98.
KOR (OPRK1)	Rhodopsin family	Dynorphin	Gαi	cAMP/CREB	Protects cartilage via ↑ anabolic enzymes and ↓ catabolism; agonists may treat early OA 101.
MC1R	Rhodopsin family - α subgroup (Melanocortin)	α-MSH	Gαs	cAMP/PKA	Reduces inflammatory cytokines and cartilage-degrading enzymes; enhances chondroprotective factors 97.
MC3R	Rhodopsin family - α subgroup (Melanocortin)	α-MSH	Gαs	cAMP/PKA	Synergizes with MC1R to suppress cartilage degradation in OA 97.
PAR2	Rhodopsin family	Proteases, e.g., trypsin	Gαq	NF-κB/ERK	Promotes OA inflammation and cartilage damage; PAR2 antagonists reduce joint swelling and senescence 94-96.

**Table 5:** The functions and mechanisms of GPCR in other cells

GPCR Name	GRAFS Classification	Ligand	Coupled G Protein Subtype	Signaling Pathway	Functional/Phenotypic Changes and references
A3AR (ADORA3)	Rhodopsin family - α subgroup (Adenosine)	Adenosine	Gαi	PKA-Akt-NF-κB inhibition	Suppresses osteosarcoma cell aggressiveness; inhibits tumor progression 124.
CaSR	Glutamate family	Extracellular Ca²⁺	Gαq	Calcium signaling/NF-κB	Promotes migration and proliferation of bone-metastasizing renal cell carcinoma (RCC) cells; potential prognostic marker for RCC bone metastasis 122.
CB1	Rhodopsin family - α subgroup (Cannabinoid)	Endocannabinoids, e.g., anandamide	Gαi	Pro-inflammatory cytokine signaling	Knockdown in Kupffer cells improves insulin sensitivity and reduces hepatic insulin resistance in obesity 125.
LPAR1 (EDG2/GPCR26)	Rhodopsin family - δ subgroup (Lipids)	Lysophosphatidic acid, LPA	Gα12/13	NF-κB/MMP activation	SNP in EDG2 promoter enhances inflammatory cytokine and MMP expression; contributes to osteoarthritis pathogenesis 126.
GPR41 (FFAR3)	Rhodopsin family	Short-chain fatty acids, e.g., propionate	Gαi/Gαq (putative)	Calcium signaling	Enhances glucose uptake in muscle cells; improves insulin sensitivity and glucose tolerance in diabetic models 123.
GPR43	Rhodopsin family	Short-chain fatty acids, e.g., propionate	Gαi	Anti-inflammatory signaling pathways	Reduce osteoarthritis cartilage inflammation and matrix destruction 103, 128.
GPRC5A	Glutamatefamily	Tretinoin	/	STAT3-dependent signaling	Knockout prevents bone metastasis in prostate cancer; correlates with Gleason score and metastasis in patients 127.
CXCR4	Rhodopsin family	CXCL12	Gαi	CXCL12/CXCR4-EMT axis	Promotes EMT-like changes and osteotropism in neuroendocrine tumor cells; CXCR4 silencing abrogates migration and metastasis 129.

**Table 6:** Drugs targeting GPCRs in clinical trials

Targeted GPCR	Drug Name	Indications	Stage (Year)	Mechanism	References
Dopamine D2 Receptor (DRD2)	Chlorpromazine	Schizophrenia	Approved (1957)	D2 antagonist	168
β1/β2-Adrenergic Receptor (ADRB1/2)	Propranolol	Hypertension, Angina, Arrhythmias	Approved (1964)	β1/β2 antagonist	169
β2-Adrenergic Receptor (ADRB2)	Salbutamol	Acute Asthma	Approved (1969)	β2 agonist	170
H1 Receptor (HRH1)	Loratadine	Allergic Rhinitis	Approved (1993)	H1 antagonist	171
Calcium-Sensing Receptor (CaSR)	Cinacalcet	Hyperparathyroidism	Approved (2004)	CaSR positive allosteric modulator	160
S1P Receptor	Fingolimod	Multiple Sclerosis	Approved (2010)	S1P1 functional antagonist	172
GLP-1 Receptor (GLP1R)	Semaglutide	T2DM, Obesity	Approved (2017)	GLP-1R agonist	173
CGRP Receptor	Erenumab (Aimovig)	Migraine Prevention	Approved (2018)	CGRP receptor antagonist (mAb)	174
GPRC5D Receptor	Teclistamab	Relapsed/Refractory Multiple Myeloma	Approved (2022)	GPRC5D antagonist (bispecific antibody)	175
5-HT1A Receptor (HTR1A)	Gepirone	Major Depressive Disorder	Approved (2023)	5-HT1A partial agonist	176
Muscarinic M1/M4 Receptor (CHRM1/4)	KarXT (Xanomeline)	Schizophrenia	Approved (2024)	M1/M4 agonist, peripheral antagonist	177
EP4 Receptor (PTGER4)	YY001 (ECNU)	Advanced Solid Tumors	Phase II (2021)	EP4 antagonist	178
CCR8 Receptor	HBM1022 (Harbour BioMed)	Solid Tumors	Phase I (2023)	CCR8 antagonist (mAb)	3
PTH1 Receptor (PTH1R)	SEP-786 (Septerna)	Hypoparathyroidism	Phase II (2023)	PTH1R oral allosteric agonist	166
GLP-1R/GCGR/GIPR	Retatrutide (Lilly)	Obesity	Phase III (2024)	Triple agonist	179
ADGRG2 Receptor	Nb23-bi (SDU)	Orchitis/Neuroinflammation	Preclinical (2025)	Allosteric nanobody (w/DHEA)	180
